# Insights into impact of polar protic and aprotic solvents on bioactive features of 3-(Dimethylaminomethyl)-5-nitroindole: A DFT study and molecular dynamics simulations

**DOI:** 10.1371/journal.pone.0330941

**Published:** 2025-09-10

**Authors:** Stève-Jonathan Koyambo-Konzapa, Berthelot S. D. Ramlina Vamhindi, Bekzod Khudaykulov, Peter A. Sidhom, Shahzeb Khan, Ashraf M. M. Abdelbacki, Alaa H. M. Abdelrahman, Mahmoud A. A. Ibrahim

**Affiliations:** 1 Laboratoire Matière, Energie et Rayonnement (LAMER), Université de Bangui, Bangui, République Centrafricaine; 2 Departement of Physics, Faculty of Science, University of Maroua, Maroua, Cameroon; 3 Department of Optics and Spectroscopy, Samarkand State University, 15 University Blvd., Samarkand, Uzbekistan; 4 Department of Pharmaceutical Chemistry, Faculty of Pharmacy, Tanta University, Tanta, Gharbia Governorate, Egypt; 5 Centre for Pharmaceutical Engineering Science, Faculty of Life Science, School of Pharmacy and Medical Sciences, University of Bradford, Bradford, United Kingdom; 6 Department of Botany and Microbiology, College of Science, King Saud University, Riyadh, Saudi Arabia; 7 Computational Chemistry Laboratory, Chemistry Department, Faculty of Science, Minia University, Minia, Egypt; 8 Department of Engineering, College of Engineering and Technology, University of Technology and Applied Sciences, Nizwa, Muscat, Sultanate of Oman; 9 School of Health Sciences, University of KwaZulu-Natal, Westville Campus, Durban, South Africa; Kwara State University, NIGERIA

## Abstract

Polar protic and aprotic solvents can effectively simulate the maturation of breast carcinoma cells. Herein, the influence of polar protic solvents (water and ethanol) and aprotic solvents (acetone and DMSO) on the properties of 3-(dimethylaminomethyl)-5-nitroindole (DAMNI) was investigated using density functional theory (DFT) computations. Thermodynamic parameters retrieved from the vibrational analysis indicated that the DAMNI’s entropy, heat capacity, and enthalpy increased with rising temperature. Natural bond orbital computations elucidated the non-bonded interactions of DAMNI. DAMNI demonstrated promising nonlinear optical properties and chemical reactivity in water, with DMSO being the second most effective solvent. Frontier molecular orbitals and global descriptors confirmed the impact of solvents on intermolecular charge transfer. The docking estimations were conducted to predict the docking pose of DAMNI against ER*α* and EGFR, both of which are acknowledged for their significance in breast cancer treatment. Upon the docking outcomes, DAMNI revealed superior binding affinity against ER*α* (calc. –5.8 kcal/mol), in comparison with DAMNI against EGFR (calc. –4.7 kcal/mol). Eventually, molecular dynamics simulations (MDS) were carried out, followed by a binding energy computation utilizing the MM/GBSA approach. Upon the MM/GBSA//150 ns MDS, the DAMNI-ER*α* complex revealed lower binding energy than DAMNI-EGFR, with Δ*G*_binding_ values of −21.1 and −15.4 kcal/mol, respectively. Post-MD analyses showed outstanding constancy of the inspected complexes over 150 ns MDS.

## Introduction

Breast carcinoma ranks among the most widespread cancers impacting women globally, representing a considerable portion of annual deaths associated with cancer [1]. In line with the World Health Organization (WHO), around 2.3 million new cases were recorded in the year 2020 [2,3]. Besides, it has been indicated that the global occurrence of breast cancer is projected to reach 28.4 million cases by 2040, representing a 47% growth since 2020 [4]. Breast cancer represents a significantly heterogeneous illness characterized by variations in the genomic, transcriptomic, proteomic, and epigenomic attributes of carcinoma cells [5]. Locoregional treatments, such as surgical and radiation therapies, represent key methods for treating breast cancer; however, contemporary standards of care now prioritize de-escalation strategies [6]. Over the last decades, treatment modalities have been selected with regard to the molecular diversity of breast cancer, placing emphasis on biologically targeted medications. Diverse systemic medications, like endocrine therapy, chemotherapy, immunotherapy, and others, have been incorporated into the curative regimen [7].

Despite considerable advancements in contemporary treatments and notable developments in scientific investigations aimed at addressing breast cancer and prolonging patient survival, a discrepancy remains in the comprehensive prevention or eradication of the disease. This is attributable to the often short-lived nature of treatment responses, the susceptibility to resistance, such as endocrine and chemotherapy resistance, or the elevated propensity for metastasis among breast cancer cells [8]. Therefore, there is an immediate necessity to investigate the molecular causal elements and a suitable therapeutic approach that can address the challenges posed by current treatment methods.

Since estrogen receptor alpha (ER*α*) predominantly governs the onset and advancement of breast cancer, it has become the foremost receptor for curative interventions in breast carcinoma disease [9,10]. Epidermal growth factor receptor (EGFR) is pivotal in modulating the advancement of breast cancer [11]. ER*α* and EGFR appear to be promising receptors for designing inhibitors used in targeted therapies developed for breast cancer [12–16].

Indole derivatives showed promise as anticancer agents, especially in developing treatments for breast cancer [17,18]. Moreover, nitro compounds can also impede the growth of breast carcinoma cells, as revealed by several previous studies [19,20]. The 3-(dimethylaminomethyl)-5-nitroindole (DAMNI) compound, also known as 5-nitrogramine, is an indole derivative that features a nitro group at the 5-position and dimethylaminomethyl groups at the 3-position [21]. Previous research has demonstrated that substituents located at the 3- and 5-positions of the indole ring significantly contribute to the enhancement of biological activities and reactivity, positioning it as a strong candidate for developing anticancer, anti-oxidant, and anti-inflammatory therapies [22–24].

Polar protic and aprotic solvents have been demonstrated to simulate metabolic maturation processes in human carcinoma cells effectively. The influence of protic solvents, such as water and ethanol, and aprotic solvents, such as DMSO, on the structural and electronic properties of the clonidine molecule was examined by S. Sarala *et al.* [25]. The water and DMSO solvents have been employed in several of our previous investigations to identify the most stable conformers and to explore the electronic properties of various molecules of interest [18,26]. More recently, our group investigated the intermolecular interactions of ethyl acetate in both aqueous and ethanolic media [27]. Additionally, numerous research facilities have established that polar solvents can suppress the proliferation of human tumors based on *in-vitro* cell assays [28]. Further preclinical investigations indicate that polar protic and aprotic solvents may serve as valuable components when used alongside standard treatment approaches [28].

This research aims to inspect the nonlinear optical (NLO) features of DAMNI, in addition to its inhibitory effects on the ER*α* and EGFR. Furthermore, the impact of aprotic solvents (acetone and DMSO) and polar protic solvents (water and ethanol) on the optimized structures, reactive sites, and the spectroscopic and NLO characteristics of DAMNI were investigated. Additionally, docking estimations were accomplished to anticipate the docking pose and score of the DAMNI against ER*α* and EGFR, which are recognized for their roles in breast cancer treatment. Finally, the DAMNI-ER*α* and DAMNI-EGFR complexes were advanced for molecular dynamics simulations (MDS), accompanied by the binding energy evaluations employing the MM/GBSA approach. The constancy of these complexes was further inspected over the 150 ns MDS. The current results highlighted the significance of evaluating DAMNI as a prospective ER*α* and EGFR inhibitor in experimental assays in order to overcome breast cancer disease.

## Computational methodology

### DFT calculations

The impact of polar protic solvents and aprotic solvents on the vibrational modes, molecular structure, active sites, and electronic features of DAMNI was evaluated. All DFT calculations were conducted utilizing the B3LYP/cc-pVTZ level using the integral equation formalism-polarizable continuum model (IEF-PCM) within the Gaussian09 software [29]. This approach provides equilibrium geometry and related vibrational properties of comparable quality to the benchmark CCSD(T) theoretical framework [30]. For DAMNI, geometrical optimization, pursued by frequency computation, was executed. VEDA 4.0 program was applied to determine the normal modes via potential energy distribution (PED) analysis [31]. Upon its optimized structure, DAMNI was submitted to a potential energy surface (PES) scan to ascertain the minimum total energies of DAMNI in different solvents. As well, the total energy (*E*_tot_) was computed by the summation of electronic energy (*E*_e_) and zero point vibrational energy (ZPVE) as follows:


$$E_{\text{tot}}=E_{\text{e}}-\text{ZPVE}$$ (1)

Upon the acquired geometries, electrostatic potential (ESP) analysis was performed, which included the creation of molecular electrostatic potential (MEP) maps utilizing an electron density envelope of 0.002 au [32]. Noncovalent interaction (NCI) analysis, a reliable tool for detecting weak noncovalent interactions [33], was performed for the investigated systems, and the three-dimensional NCI plots were illustrated using color-coded isosurfaces. NCI computations were carried out utilizing the Multiwfn 3.7 software [34] and portrayed through the visual molecular dynamics (VMD) package [35].

Furthermore, frontier molecular orbitals (FMOs) theory was inspected for DAMNI to comprehend its electronic properties. From FMOs, the maps and energies of the highest occupied/lowest unoccupied molecular orbitals (HOMO/LUMO) were examined. Subsequently, the energy gap (*E*_gap_) was determined based on the *E*_HOMO_ and *E*_LUMO_ values as outlined below:


$$E_{\text{gap}}=E_{\text{LUMO}}-E_{\text{HOMO}}$$ (2)

Moreover, the electron affinity (*EA*) and ionization potential (*IP*) were computed based on *E*_LUMO_ and *E*_HOMO_, respectively, in the following manner:


$$\;\;\;\;\;\;\;\;\;\;\;\;\;\;\;\;\;\;\;\;\;\;\;\;\;\;\;\;\;\;\;\;\;\;\;\;\;\;\;\;\;\;\;\;\;\;\;\;\;\;\;\;\;\;\;\;\;EA\approx -E_{LUMO}$$ (3)

$$\;\;\;\;\;\;\;\;\;\;\;\;\;\;\;\;\;\;\;\;\;\;\;\;\;\;\;\;\;\;\;\;\;\;\;\;\;\;\;\;\;\;\;\;\;\;\;\;\;\;\;\;\;IP\approx -E_{HOMO}$$ (4)

The calculations for chemical potential (*µ*), electrophilicity index (*ω*), global softness (*S*), electronegativity (*χ*), and global hardness (*η*) were performed employing the subsequent equations:


$$\eta =\frac{E_{\text{LUMO}}-E_{\text{HOMO}}}{2}$$ (5)

$$\mu =\frac{E_{\text{LUMO}}+E_{\text{HOMO}}}{2}$$ (6)

$$S=\;\frac{1}{\eta }$$ (7)

$$\omega =\;\frac{\mu^{2}}{2\eta }$$ (8)

χ= −μ (9)

Based on electronic representation, the density of states (DOS) evaluation was conducted for DAMNI employing GaussSum software [36]. Accordingly, the partial density of states (PDOS) plot was illustrated.

In the context of frequency computations, thermodynamic parameters were assessed for DAMNI. Consequently, the changes in Gibbs free energy (Δ*G*), entropy (Δ*S*), and enthalpy (Δ*H*) could be determined as follows:


ΔS=−(ΔG− ΔH)/T (10)

Furthermore, Raman and infrared (IR) spectroscopic analyses were conducted on the examined DAMNI. To ascertain the characteristics of the interactions, NBO calculations were performed utilizing the NBO 3.1 program implemented inside the Gaussian09 software [37]. In that spirit, the stabilization interaction energy, *E*(2), was determined through second-order perturbation theory [38], as follows:


$$\;E(2)=\Delta E_{ij}=q_{i}\frac{F_{\left(i,j\right)}^{2}}{\varepsilon_{i}-\varepsilon_{j}}$$ (11)

Here, $q_{i}$ and $F_{\left(i,j\right)}$ represent the donor occupancy and the off-diagonal elements of the NBO Fock matrix, respectively. $\varepsilon_{i}$ and $\varepsilon_{j}$ denote the diagonal elements of the matrix. The VMD software was applied to display the color-filled reduced density gradient (RDG) isosurface, while Multiwfn software generated a localized orbital locator (LOL) and electron localization function (ELF). The RDG analysis is based on the electronic density ρ(r) and its gradient ∇ρ(r), as outlined below:


$$\;RDG(r)=\frac{1}{2{\left(3\pi^{2}\right)}^{\frac{1}{3}}}\frac{\left|\nabla \rho (r)\right|}{{\rho (r)}^{\frac{4}{3}}}$$ (12)

Nonlinear optical (NLO) properties of DAMNI were estimated. The first-order hyperpolarizability is an essential parameter for determining the NLO properties of organic materials. First-order hyperpolarizability refers to a third-rank tensor characterized by the coefficients present in the Taylor series expansion of energy concerning the external electric field [39]. If the external electric field is weak and homogeneous, the energy expansion is given by:


$$E=E^{0}-1/2a_{ab}F_{a}F_{b}-m_{a}F_{a}-1/6b_{abg}F_{a}F_{b}F_{g}+...$$ (13)

where *μ*_*α,*_*β*_*αβγ*_,and *α*_*αβ*_ are the components of dipole moment, the first hyperpolarizability and polarizability, respectively. *E*^*0*^ denotes the energy of the unperturbed molecules, while *F*_*α*_corresponds to the applied electric field. The dipole moment (*μ*), static polarizability (*α*_0_), anisotropy of the polarizability (Δ*α*)*,* and first-order hyperpolarizability (*β*) are defined as [40]:


$$\mu =(\mu_{x}+\mu_{z}+\mu_{y}{)}^{1/2}$$ (14)

$$\alpha_{0}=1/3(\alpha_{xx}+\alpha_{zz}+\alpha_{yy})$$ (15)

$$\Delta \alpha =2^{-1/2}[(\alpha_{xx}-\alpha_{yy}{)}^{2}+(\alpha_{zz}-\alpha_{xx}{)}^{2}+(\alpha_{yy}-\alpha_{zz}{)}^{2}+6{\alpha^{2}}_{xz}{]}^{1/2}$$ (16)

$$\beta =((\beta_{xxx}+\beta_{xyy}+\beta_{xzz}{)}^{2}+(\beta_{zzz}+\beta_{xxz}+\beta_{yyz}{)}^{2}+(\beta_{yyy}+\beta_{xxy}+{\beta_{yzz}}^{)2}{)}^{1/2}$$ (17)

### Molecular docking

All docking calculations presented in this study were conducted utilizing the AutoDock4.2.6 software [41]. The three-dimensional structures of wild ER*α* and EGFR (PDB IDs: 3ERT [42] and 5WB7 [43], respectively) were sourced from the RCSB-PDB [44]. For the sake of preparation of the investigated proteins, crystallographic water, ions, heteroatoms, and ligands were removed. Besides, the PROPKA software was employed to assign the ionization state of the titrable amino acids of the investigated targets [45]. Afterward, all missing hydrogen atoms were inserted.

The DAMNI chemical structure underwent minimization employing the MMFF94S force field implemented within SZYBKI software [46,47]. The atomic charge of DAMNI was subsequently assigned using the Gasteiger-Marsili method [48].

Prior to executing docking calculations, the examined targets were transformed into pdbqt format utilizing MGL1.5.6 tools [49]. Genetic algorithm (*GA*) run and energy evaluations (*eval*) were adjusted to 250 and 25,000,000, respectively. The default values of the rest docking settings were used. A grid box measuring 50 Å × 50 Å × 50 Å in the *x*, *y*, and *z* dimensions was employed to encompass the active sites of ER*α* and EGFR. The grid maps, featuring a spacing of 0.375 Å, were generated utilizing the AutoGrid software [50]. The grid was positioned at the core of the active sites of ER*α* and EGFR. Molecular interactions were depicted using the Discovery Studio Visualizer [51].

### Molecular dynamics simulations (MDS)

AMBER20 software was used to conduct MDS for DAMNI-ER*α* and DAMNI-EGFR complexes [52]. A detailed description of the MDS parameters can be found in Refs [53–56]. Concisely, the DAMNI was characterized by employing General AMBER Force Field (GAFF2) [57]. As well, the AMBER force field 14SB was employed to parameterize ER*α* and EGFR targets [58]. The restricted electrostatic potential (RESP) approach was employed to determine the atomic charges of the DAMNI at the HF/6-31G* level using Gaussian09 software [59]. The docked DAMNI-ER*α* and DAMNI-EGFR complexes were placed within a truncated octahedral box of TIP3P water molecules measuring 12 Å [60]. The solvated complexes were neutralized by adding Cl^−^ or Na^+^ ions. The ionic strength of the solution was maintained at 0.15 M NaCl. The examined complexes were initially subjected to energetic minimization for 5000 cycles. The minimized complexes were incrementally warmed from 0 to 310 K over a brief duration of time (50 ps) under the NVT conditions. The heated complexes were then equilibrated over 10 ns under NPT conditions. The production phase for each complex was subsequently conducted for a duration of 150 ns under NPT conditions. MDS was performed utilizing the CUDA version of the pmemd module within AMBER20 software.

### Binding energy computations

The binding energy (Δ*G*_binding_) of the studied complexes was evaluated using the molecular mechanics/generalized Born surface area (MM/GBSA) approach [61]. The calculations of Δ*G*_binding_ were performed on uncorrelated trajectories obtained from the MDS. The subsequent equation was then employed to evaluate the Δ*G*_binding_.


$$\;{\Delta G}_{\text{binding}}=G_{\text{Complex}}-G_{\text{Target}}\;-G_{\text{DAMNI}}$$ (18)

The energy (*G*) term is obtained through the subsequent mathematical equation:


$$\;G=G_{\text{SA}}+G_{\text{GB}}+E_{\text{ele}}+E_{\text{vdW}}$$ (19)

*G*_GB_ and *G*_SA_ represent polar and nonpolar solvation-free energy, respectively. *E*_vdW_ and *E*_ele_ denote the van der Waals interactions and the electrostatic forces, respectively. Due to the significant computational expense, entropy effects were not considered [62,63].

## Results and discussion

### ESP analysis

**Fig 1:**
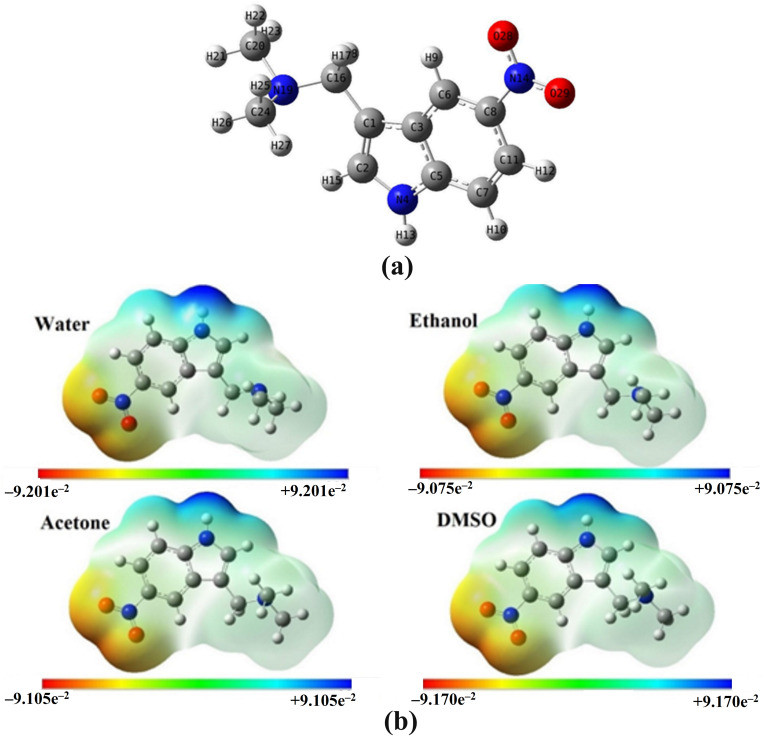
(a) Optimized geometric structure of DAMNI with atom numbering and (b) MEP maps of DAMNI in polar protic and aprotic solvents. Different colors are employed to indicate the distribution of charge, specifically blue, red, and green, which signify positive charge, negative charge, and neutral charge, respectively. The blue area is characterized by a low electron density and weak interactions, whereas the red area exhibits a high electron density and significant potential for interactions.

Herein, an analysis of ESP was employed to carefully distinguish the nucleophilic and electrophilic nature of DAMNI in different solvent environments, and the MEP maps were generated. Fig 1 depicts the MEP maps of DAMNI in various solvents. As previously noted, the presence of solvents increases the number of reactive sites on a molecule compared to the gas phase [25]. In each solvation model, the O atoms in the NO_2_ group of DAMNI displayed negative regions (red), while the H atoms in the NH group of the pyrrole ring and the amine group showed positive regions (blue). Thus, O28 and O29 were identified as the primary sites for electrophilic reactions, and H13 was the most likely site for nucleophilic attacks. Electron-deficient areas were noticed on the methyl and methylene groups attached to N19, as well as on the regions between components of the indole group. As illustrated in Fig 1, the MEP values ranged from –9.201e^–2^ to +9.201e^–2^ au in water, from –9.075e^–2^ to +9.075e^–2^ au in ethanol, from –9.105e^–2^ to +9.105e^–2^ au in acetone, and from –9.170e^–2^ to +9.170e^–2^ au in DMSO.

### PES scan

**Fig 2:**
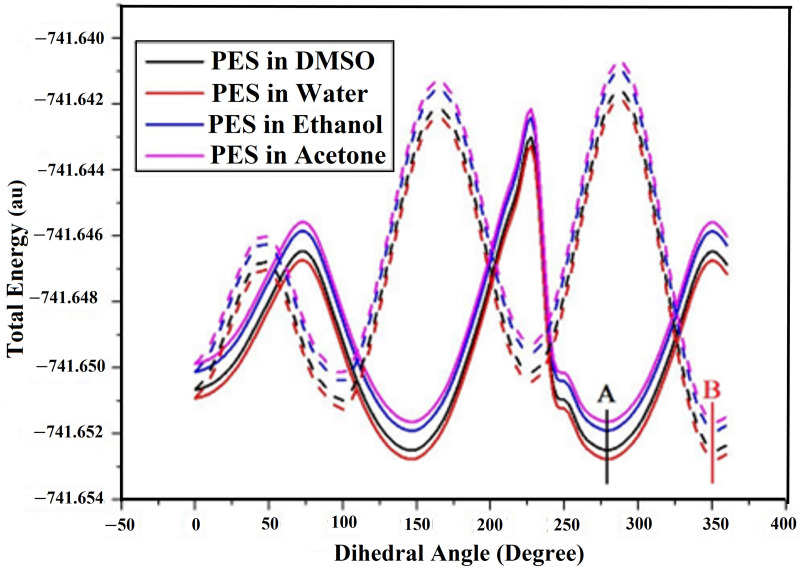
PES scans of DAMNI considering the H17-C16-C1-C2 dihedral angle (solid line) and the C20-N19-C16-C1 dihedral angle (dashed line) in polar protic and aprotic solvents. **A** and **B** indicate the points corresponding to the global minima obtained after a complete 360-degree rotation of the dihedral angles.

A PES scan was devoted to recognizing the lowest total energy of DAMNI in various solvents. These studies involved rotating the H17-C16-C1-C2 and C20-N19-C16-C1 angles by 360 degrees. The PES scan calculations were conducted utilizing the B3LYP/cc-pVTZ method in water, ethanol, acetone, and DMSO (Fig 2). The letters **A** and **B** in Fig 2 indicate the points corresponding to the global minima obtained after a complete 360-degree rotation of the dihedral angles H17-C16-C1-C2 and C20-N19-C16-C1, respectively. The lowest energy values associated with points **A** and **B** were –741.65277 and –741.65275 au in water, –741.65190 and –741.65188 au in ethanol, –741.65163 and –741.65162 au in acetone, and –741.65249 and –741.65248 au in DMSO. The angle values associated with these minimum energies in the studied solvents, as well as the details on other conformations, are provided comprehensively in S1 Table. The parameters related to the most stable molecular configuration of DAMNI, encompassing *E*e, ZPVE, and *E*_tot_, are presented in Table 1. The minimum energy values of the optimized molecular geometries were determined to be −741.654, −741.653, −741.653, and −741.654 au in water, ethanol, acetone, and DMSO, respectively. However, the lowest energy values for DAMNI are observed in water and DMSO as the surrounding environments. S2 Table provides the geometric parameters, like bond angles, dihedral angles, and bond lengths. According to S2 Table, the bond lengths were nearly identical, which can be ascribed to the minimal impact of both polar protic and aprotic solvents on these geometric parameters. A slight influence was, however, noted on the bond involving the C8 atom, directly connected to the NO_2_ group. In contrast, the effect of the different solvents was more apparent on the dihedral angles of DAMNI.

**Table 1:** The computed *E*_e_, ZPVE, and *E*_tot_ of DAMNI in polar protic and aprotic solvents.

Energy (au)	Solvent			
	Water	Ethanol	Acetone	DMSO
*E*_e_	−741.886	−741.886	−741.885	−741.886
ZPVE	0.233	0.233	0.233	0.233
*E*_tot_	−741.654	−741.653	−741.653	−741.654

### NCI-RDG analysis

**Fig 3:**
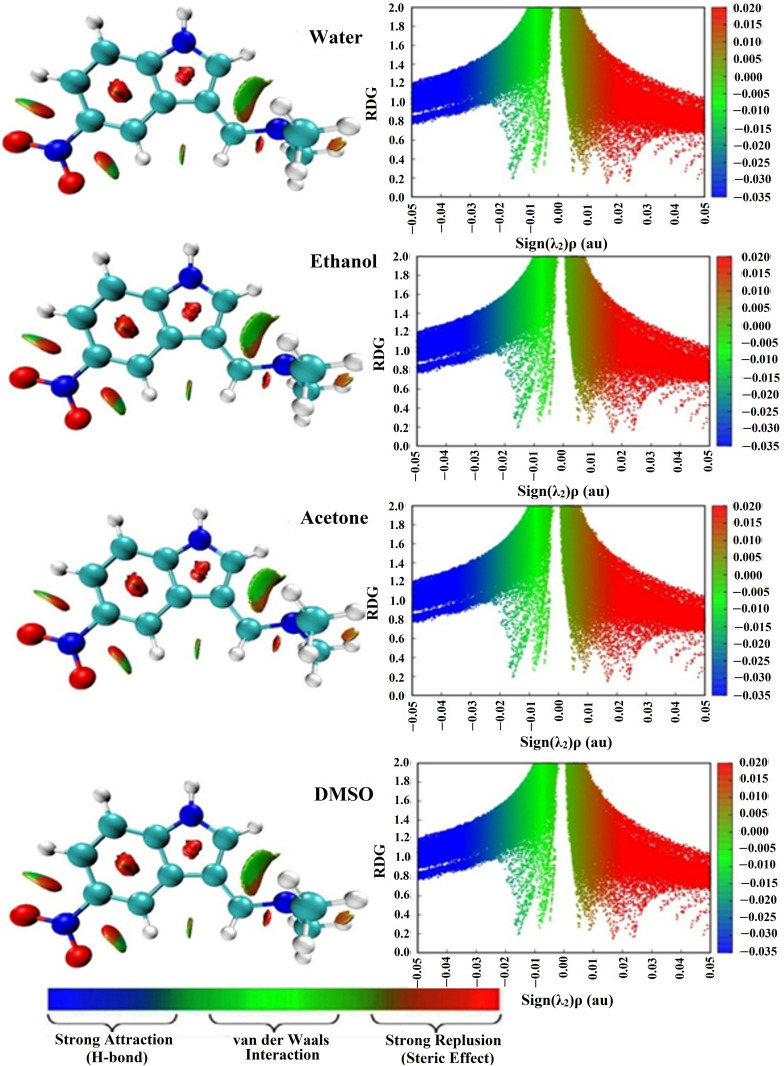
NCI plots of DAMNI in polar protic and aprotic solvents. The 2D RDG isosurfaces were graphed at an RDG value of 0.50 au and illustrated with sign(λ_2_)ρ values along a color gradient from −0.035 (blue) to 0.020 (red) au.

NCI analysis was considered a reliable tool to provide in-depth insights into the nature of the interactions of the DAMNI in various solvents. The corresponding NCI isosurface plots were created and are illustrated in Fig 3. NCI plots demonstrated the occurrence of strong repulsive interactions between the aromatic rings of DAMNI across all solvent models ranging from 0.01 to 0.05 au. As well, the NCI plots revealed vdW interactions within DAMNI ranging from −0.01 to 0.01 au. The blue regions suggested that there were no strong attractions (such as H-bonds) in DAMNI. This analysis showed that the 3D isosurface and 2D RDG plots of DAMNI were not significantly affected by the surrounding environment.

### LOL and ELF analyses

**Fig 4:**
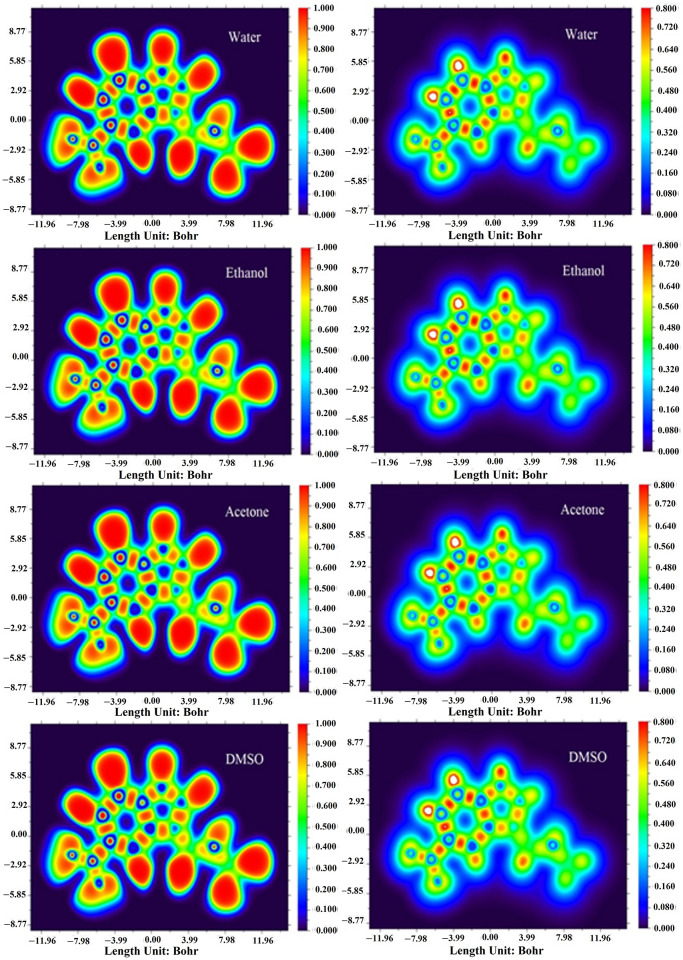
ELF and LOL plots of DAMNI within polar protic and aprotic solvents. The color code: cyan for domains with lone pairs, red for domains associated with hydrogen atoms, and blue for bonding domains that involve heavy atoms.

The LOL and ELF analyses were performed to detect the electron density at the antibonding and bonding sites of the DAMNI in various solvents. ELF considers the density of electron pairs, while LOL is solely concerned with the gradients of localized orbitals [64]. Fig 4 displays the contour maps and color shade of the LOL and ELF for DAMNI in various solvents. The color scale on the *y*-axis ranged from blue to red, with ELF values spanning from 0 to 1.0 and LOL values from 0 to 0.8. Values greater than 0.5 indicate regions of electron localization, while values below 0.5 signify areas with delocalized electrons. The red color corresponds to higher ELF (or LOL) values, while the blue color indicates lower ELF (or LOL) values. From Fig 4, the red areas around the H-atoms represented the highest ELF or LOL values in DAMNI, suggesting a high electron density from covalent bonding, such as from a nuclear shell or lone electron pair. However, the blue regions over the nitrogen and carbon atoms reflected lower electron localization, indicating electron-deficient areas between the inner and valence shells. Additionally, small white circles around specific hydrogen atoms in the LOL maps near the benzene ring highlighted regions where electron density exceeded the upper limit (values greater than 0.8).

### NBO analysis

NBO analysis serves as an essential method for investigating intramolecular H-bonding, intramolecular charge transfer (ICT), and interactions related to bonding. NBO analysis, using the *E*(2), facilitates the examination of both occupied and unoccupied orbitals, providing deeper insights into intermolecular hyperconjugation interactions, H-bond establishment, and ICT within the molecule [65]. Table 2 highlights the significant delocalization of donor (i) to acceptor (j) orbitals and the *E*(2) for DAMNI in water, as computed utilizing second-order Fock matrix perturbation theory.

**Table 2:** Second-order perturbation analysis of Fock matrix in NBO basis of DAMNI in water.

No.	Donor NBO (i)	ED (e)	Acceptor NBO (j)	ED (e)	*E*(2) (kcal/mol)	*E* (j) − *E* (i) (au)	F(i,j) (au)
1.	σ (C1 – C2)	1.97560	σ*(C3 – C6)	0.02018	6.45	1.25	0.08
2.	π (C1 – C2)	1.82882	π*(C3 – C5)	0.51520	16.62	0.28	0.067
3.	σ (C2 – N4)	1.98446	σ*(C5 – C7)	0.02075	5.46	1.32	0.076
4.	σ (C3 – C5)	1.96250	σ*(C1 – C16)	0.02344	5.49	1.06	0.068
5.	π (C3 – C5)	1.52811	π* (C1 – C2)	0.27531	17.39	0.28	0.066
6.	π (C3 – C5)	1.52811	π* (C6 – C8)	0.39139	26.10	0.26	0.075
7.	π (C3 –C5)	1.52811	π* (C7– C11)	0.28345	16.33	0.27	0.062
8.	σ (C6 – C8)	1.97598	σ*(C1 – C3)	0.02488	5.14	1.22	0.071
9.	π (C6 – C8)	1.67870	π* (C3– C5)	0.51520	14.35	0.29	0.06
10.	π (C6 – C8)	1.67870	π* (C7– C11)	0.28345	19.32	0.29	0.068
11.	π (C6 – C8)	1.67870	π*(N14– O29)	0.65507	37.98	0.13	0.068
12.	σ (C6 – H9)	1.67428	σ*(C8 – C11)	0.02231	5.00	1.03	0.064
13.	σ (C7 – H10)	1.97404	σ* (C3 – C5)	0.02917	5.22	1.01	0.065
14.	σ (C7 – C11)	1.97535	σ*(N4 – C5)	0.02353	5.77	1.17	0.073
15.	π (C7 – C11)	1.73675	π* (C3 – C5)	0.51520	19.17	0.28	0.07
16.	π (C7 – C11)	1.73675	π* (C6 – C8)	0.39139	16.74	0.28	0.063
17.	σ (C11 – H12)	1.97275	σ* (C6 – C8)	0.01945	5.12	1.05	0.066
18.	π (N14 – O29)	1.98738	LP (3) O28	1.49230	10.93	0.17	0.075
19.	π (N14 – O29)	1.98738	π* (N14 – O29)	0.65507	7.76	0.31	0.053
20.	σ (C16 – H18)	1.96653	π*(C1– C2)	0.27531	5.22	0.52	0.049
21.	LP (1) N4	1.58793	π*(C1 – C2)	0.27531	33.15	0.30	0.092
22.	LP (1) N4	1.58793	π*(C3 – C5)	0.51520	43.41	0.28	0.101
23.	LP (1) N19	1.87587	σ*(C16 – H17)	0.03695	7.49	0.66	0.064
24.	LP (1) N19	1.87587	σ*(C20 – H22)	0.03033	7.75	0.66	0.065
25.	LP (1) N19	1.87587	σ*(C24 – H25)	0.02969	7.56	0.66	0.064
26.	LP (2) O28	1.90400	σ*(C8 – N14)	0.10151	13.47	0.59	0.079
27.	LP (2) O28	1.90400	σ*(N14 – O29)	0.05726	19.02	0.70	0.104
28.	LP (2) O29	1.90495	σ*(C8 – N14)	0.10151	13.32	0.59	0.079
29.	LP (2) O29	1.90495	σ*(N14 – O28)	0.05723	19.03	0.70	0.104

S3 Table provides additional NBO information obtained for DAMNI in DMSO, acetone, and ethanol solvents. Strong inter- and intra-molecular interactions were identified in electronic transitions involving π → π*, σ → σ*, and lone pair (LP) → π* (or σ*) transitions. In general, the ICT interactions between lone pair electrons and antibonding π orbitals play a fundamental function in the molecule’s biological activity. Table 2 and S3 Table highlight high *E*(2) values for the lone pair (LP) electrons of N4 transitioning to the π* (C3–C5) orbitals, with interaction energies of 43.41, 43.19, 43.13, and 43.34 kcal/mol in water, ethanol, acetone, and DMSO, respectively. The highest second-order perturbation energy measured at 43.41 kcal/mol for water, as indicated by the NBO analysis, was associated with the electron delocalization from the donor LP (1) N4 to the π*(C3 – C5) acceptor interaction.

Conversely, ICT interactions between bonding and antibonding π orbitals were generally associated with the highest stabilization, contributing significantly to the molecule’s nonlinear optical (NLO) properties. For the π (C6–C8) to π* (N14–O29) transitions, *E*(2) interaction energy values of 37.98, 37.51, 37.37, and 37.83 kcal/mol were observed in water, ethanol, acetone, and DMSO, respectively. The results indicated that *E*(2) interaction energy values increased as the dielectric constant of the solvent environment rose.

### Polarizability and first-order hyperpolarizability

NLO materials are commonly employed in optical apparatus, including optical switches, modulators, communication systems, and data storage technologies [43]. Aromatic compounds containing nitrogen, such as indole derivatives, are typically more effective for NLO activities owing to the existence of lone pairs of electrons [41]. The NLO properties of a system, along with the magnitude of molecular interactions, including long-range induction, dispersion forces, and so forth, are determined by its polarizabilities and hyperpolarizabilities. Furthermore, these properties also affect the cross-sections associated with different collision and scattering events. The dipole moment, first-order hyperpolarizability, and polarizability of DAMNI in different solvation models were computed utilizing the B3LYP/cc-pVTZ method, as illustrated in Table 3. The total dipole moment (*μ*) for the DAMNI molecule was found to be 9.907, 9.778, 9.738, and 9.866 Debye in water, ethanol, acetone, and DMSO, respectively. The static polarizability (*α*0) and anisotropy of polarizability (Δ*α*) for DAMNI were observed with values of 242.137 and 181.691 au in water, 238.584 and 178.085 au in ethanol, 237.504 and 177.004 au in acetone, and 241.011 and 180.540 au in DMSO, respectively. The first-order hyperpolarizability (*β*) for the molecule was estimated to be 450.9x10 ⁻ ³¹, 424.6x10 ⁻ ³¹, 416.8x10 ⁻ ³¹, and 442.5x10 ⁻ ³¹ esu in water, ethanol, acetone, and DMSO, respectively, compared to the urea molecule, a reference compound with a value of 6.56x10 ⁻ ³¹ esu. The *β*xzz components mainly contributed to these hyperpolarizability values, indicating significant charge delocalization along this axis. Moreover, the dipole moment was active in the *z*-direction in all solvents. These results showed that the dipole moment, first-order hyperpolarizability, and polarizability increased with the solvent’s dielectric constant, as observed in several prior studies [66,67]. Consequently, DAMNI exhibited enhanced NLO properties in water, followed by DMSO as the surrounding solvent.

**Table 3:** Dipole moment, first-order hyperpolarizability, and polarizability of DAMNI in polar protic and aprotic solvents.

Parameters	Water	Ethanol	Acetone	DMSO	Parameters	Water	Ethanol	Acetone	DMSO
***Dipole moment (μ*) *in Debye (D)***					***First-order hyperpolarizability (β*) *in au***				
*μ*_x_	−4.903	−4.848	−4.831	−4.886	*β*_xxx_	−34.148	−42.368	−44.769	−36.807
*μ*_y_	0.159	0.156	0.155	0.158	*β*_xxy_	32.197	30.599	30.122	31.685
*μ*_z_	8.607	8.490	8.454	8.570	*β*_xyy_	−126.066	−123.266	−122.422	−125.176
*μ*	9.907	9.778	9.738	9.866	*β*_yyy_	72.117	71.154	70.855	71.813
***Polarizability (α*) *in au***					*β*_xxz_	−243.969	−228.234	−223.580	−238.927
*α*_xx_	262.611	259.625	258.710	261.669	*β*_xyz_	−8.860	−8.233	−8.046	−8.657
*α*_xy_	1.579	1.423	1.375	1.530	*β*_yyz_	47.024	45.445	44.980	46.518
*α*_yy_	132.864	131.291	130.809	132.368	*β*_xzz_	738.734	700.600	689.008	727.167
*α*_xz_	−29.405	−29.323	−29.299	−29.379	*β*_yzz_	210.608	198.619	195.077	206.743
*α*_yz_	−3.789	−3.568	−3.502	−3.719	*β*_zzz_	−4980.91	−4693.73	−4608.96	−4888.47
*α*_zz_	330.935	324.835	322.992	328.995	*β*	5219.691	4914.961	4825.003	5121.621
*α*_0_	242.137	238.584	237.504	241.011	*β*in esu	450.9x10^−31^	424.6x10^-31^	416.8x10^−31^	442.5x10^−31^
Δ*α*	181.691	178.085	177.004	180.540		(1 au = 8.6393x10^−33^ esu)			

### Frontier molecular orbitals and global descriptors

**Fig 5:**
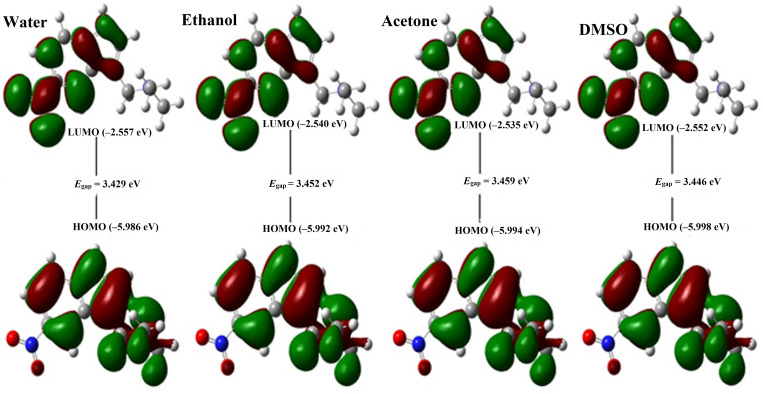
Visualization of the FMOs for DAMNI in polar protic and aprotic solvents.

Toward electronic insights of DAMNI, the FMOs theory was applied. Within FMOs, the LUMO and HOMO energy levels were graphed for the investigated DAMNI molecule in different solvents and are plotted in Fig 5. The *E*_HOMO_, *E*_LUMO_, and *E*_gap_ values were computed and are compiled in Table 4.

**Table 4:** Estimated the quantum mechanical parameters of DAMNI in polar protic and aprotic solvents.

Solvent	*E*_HOMO_ (eV)	*E*_LUMO_ (eV)	*E*_gap_ (eV)	*IP* (eV)	*EA* (eV)	*χ* (eV)	*η* (eV)	*S* (eV^–1^)	*μ* (eV)	*ω* (eV)
**Water**	–5.986	–2.557	3.429	5.986	2.557	4.272	1.715	0.583	–4.272	5.321
**Ethanol**	–5.992	–2.540	3.452	5.992	2.540	4.266	1.726	0.579	–4.266	5.272
**Acetone**	–5.994	–2.535	3.459	5.994	2.535	4.265	1.729	0.578	–4.265	5.258
**DMSO**	–5.998	–2.552	3.446	5.998	2.552	4.275	1.723	0.580	–4.275	5.303

It is noteworthy that the distributions of HOMO and LUMO were predominantly situated in the regions of electronegativity and electropositivity, respectively. For DAMNI in different solvents, the patterns of the HOMO and LUMO were concentrated over nucleophilic sites (i.e., N and O of the NO_2_ group) and electrophilic sites (i.e., H and C of the pyrrole ring and the amine group), respectively.

As shown in Table 4, *E*_HOMO_ and *E*_LUMO_ for DAMNI within various solvents revealed distinct values, indicating the influence of the solvent on its electronic nature. Numerically, DAMNI in water, ethanol, acetone, and DMSO demonstrated *E*_gap_ with values of 3.429, 3.452, 3.459, and 3.446 eV, respectively. A smaller energy gap facilitates ICT, which may enhance the molecule’s polarity and interaction potential. Upon the estimated *E*_gap_ values, the DAMNI demonstrated the subsequent sequence of chemical reactivity in different solvents: water > DMSO > ethanol > acetone.

In accordance with the unequivocal significance of electronic parameters, global reactivity indices were computed for DAMNI in various solvents. The *IP* was found with values of 5.986, 5.992, 5.994, and 5.998 eV for DAMNI within water, ethanol, acetone, and DMSO, respectively. The hardness (*η*) of DAMNI within different solvents can also serve as an indicator of its stability and reactivity. Numerically, *η* was found to be 1.715, 1.726, 1.729, and 1.723 eV for DAMNI within water, ethanol, acetone, and DMSO, respectively. The softness (*S*) degree of DAMNI within various solvents represents one of the critical factors affecting its steadiness and reactivity. The *S* of DAMNI in various solvents ascended in the following order: water > DMSO > ethanol > acetone (Table 4).

### DOS analysis

**Fig 6:**
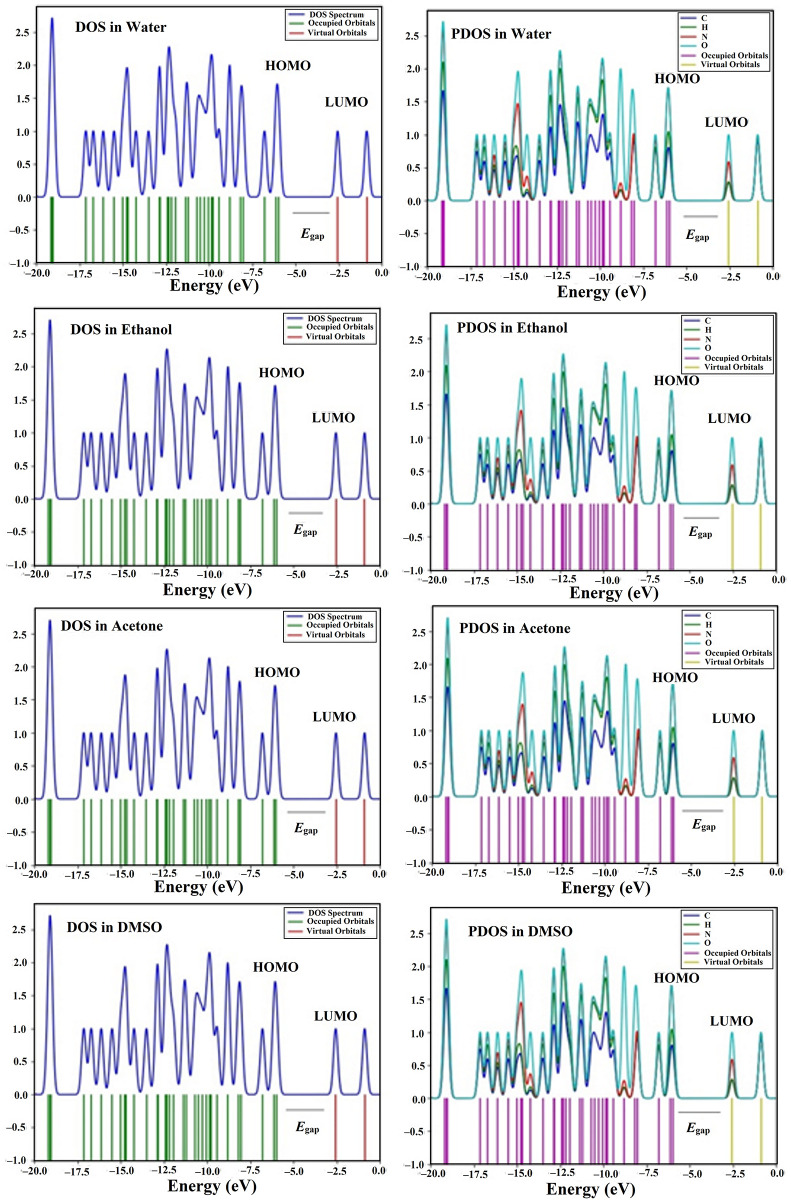
DOS and PDOS plots of DAMNI in polar protic and aprotic solvents.

For additional electronic characteristics of DAMNI in different solvents, the density of states (DOS) analysis was accomplished. Fig 6 displays the DOS and partial density of states (PDOS) spectra of DAMNI in various solvent environments. The DOS and PDOS evaluations support the FMOs analysis by offering significant perspectives regarding the properties of the molecular orbitals (MO) as well as the contributions from various groups within DAMNI, taking into account their surrounding environment. Significant DOS or PDOS intensity at certain energy levels indicated the presence of occupied states, while a lack of intensity signified the absence of occupied states.

### Thermodynamic parameters

**Fig 7:**
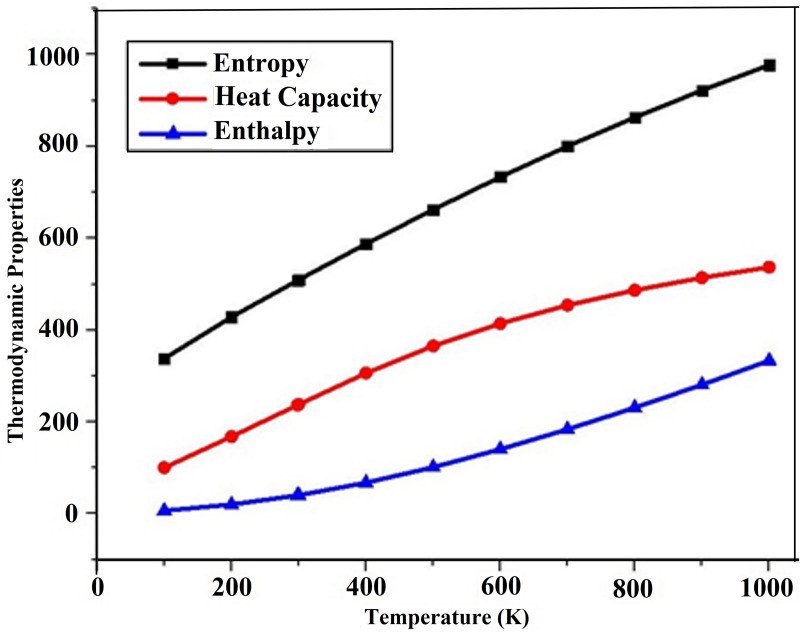
Thermodynamic parameters of DAMNI in water solvent.

In the pursuit of thermal understanding, thermodynamic properties were evaluated for DAMNI in various solvent environments and temperatures ranging from 100 to 1000 K. This analysis provides significant understanding regarding the reactivity, stability, and behavior of the molecule in various conditions. Considering temperature is essential in chemical thermodynamics and pharmacodynamics, physicochemical effects significantly impact industries such as food preservation and pharmaceutical storage [68]. S4 Table outlines the thermodynamic parameters considered in this study, including the calculated heat capacity at constant pressure (Cp), enthalpy change (Δ*H*), and entropy (*S)*, with the corresponding graph in water presented in Fig 7. As shown in S4 Table and Fig 7, Cp, *S*, and Δ*H* increased with temperature, which can be ascribed to the increased molecular vibrational intensities as the temperature rises.

### Raman and IR spectra

**Fig 8:**
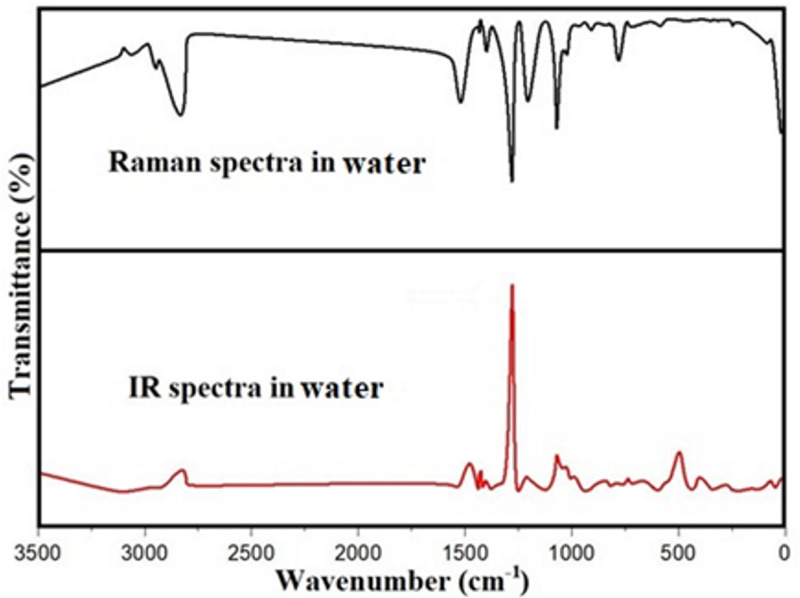
Theoretical IR and Raman spectra of DAMNI in water solvent.

Raman and IR spectroscopy are essential tools for analyzing molecular vibrations, helping to confirm the most stable optimized structure and identify various functional groups [69]. DAMNI, composed of 29 atoms, exhibits 81 typical vibrational modes and features a non-planar configuration within the C1 symmetry group. Given the lack of experimental data on DAMNI, this study employs the IR and Raman spectral data reported by S. Christopher *et al.* [70], with a specific focus on the nitroindole group. The simulated scaled wavenumbers for the four solvents utilized in this investigation, including IR intensities, Raman activities, and the classification of vibrational normal modes based on potential energy distribution (PED) values in water, are displayed in S5 Table. A scaling factor of 0.9619 was utilized to rectify limitations associated with the basis set and to address vibrational inconsistencies [71]. The theoretical Raman and IR spectra of DAMNI in water are shown in Fig 8, while those for DMSO, acetone, and ethanol are provided as supplementary information in S1 Fig.

It has been shown that the symmetric stretching modes of the NO_2_ group appear between 1370 and 1320 cm^–^^1^, while the asymmetric stretching modes were noticed between 1570 and 1485 cm^–^^1^. As indicated in S5 Table, the wavenumber values for the symmetric stretching mode of the NO_2_ group in DAMNI, obtained through simulations, were 1270.43, 1270.27, 1270.22, and 1270.38 cm^–^^1^ for water, ethanol, acetone, and DMSO, respectively. These calculated values were not detected in the existing Raman and IR spectra. The wavenumber values falling outside the expected range can be elucidated by the impact of the NO_2_ group’s bending mode coupled with its stretching mode. The absorption peaks for the asymmetric stretching modes of the NO_2_ group were noticed at 1436 and 1393 cm^–^^1^ in both IR and Raman spectra. The calculated frequencies for these modes were 1440.32 and 1416.60 cm^–^^1^ in water, 1440.82 and 1417.00 cm^–^^1^ in ethanol, 1440.97 and 1417.13 cm^–^^1^ in acetone, and 1440.47 and 1416.73 cm^–^^1^ in DMSO. The bending modes of the NO_2_ group in DAMNI were calculated, yielding values of 813.63, 751.48, 607.39, and 493.21 cm^–^^1^ in water. Some of these modes were experimentally monitored in the IR and Raman spectra, as shown in S5 Table, which also included the calculated wavenumber values obtained in the other solvents used in this study.

The NH group is bound to the pyrrole component of the indole structure. This group typically showed a notable PED in its stretching vibration mode, with wavenumbers generally in the range of 3500–3450 cm^–^^1^ [72]. The NH peak appeared at 3460 cm^–1^ on the IR spectrum, while the calculated values for water, ethanol, acetone, and DMSO were 3495.83, 3497.23, 3497.67, and 3497.28 cm^–^^1^, respectively, with a 100 percent PED contribution. The bending and torsion modes, including the NH group, were also recorded and provided in S5 Table. The CC group stretching modes appeared in the IR (Raman) spectra at 1578 (1577), 1517 (1518), 1456 (1456), 1436 (1436), and 1295 cm^–^^1^ (1299 cm^–^^1^). The corresponding calculated vibrational frequencies in water were 1546.21, 1519.95, 1474.60, and 1440.32 cm^–^^1^. All these wavenumber values fell within the expected range, typically between 1650 and 1400 cm^–^^1^ [73].

The CH stretching vibrations of heteroaromatic compounds generally appear in the 3125–3000 cm^–^^1^ range [70]. In DAMNI, the computed frequencies for the CH stretching modes of the indole group were found between 3137.93–3071.89 cm^–^^1^ in water, 3137.87–3071.45 cm^–^^1^ in ethanol, 3137.86–3071.32 cm^–^^1^ in acetone, and 3137.91–3071.76 cm^–^^1^ in DMSO. Additional calculated frequencies corresponding to CH groups throughout DAMNI were observed in water from 2810.80 to 1474.60 cm^–^^1^, in ethanol from 2810.79 to 1476.05 cm^–^^1^, in acetone from 2810.79 to 1476.50 cm^–^^1^, and in DMSO from 2810.80 to 1475.05 cm^–^^1^. Some of these theoretical frequencies were matched with peaks identified in the IR and Raman spectra, with the corresponding wavenumbers provided in S5 Table. The normal vibrational modes associated with methyl and methylene groups are also reported in S5 Table.

### Molecular docking

**Fig 9:**
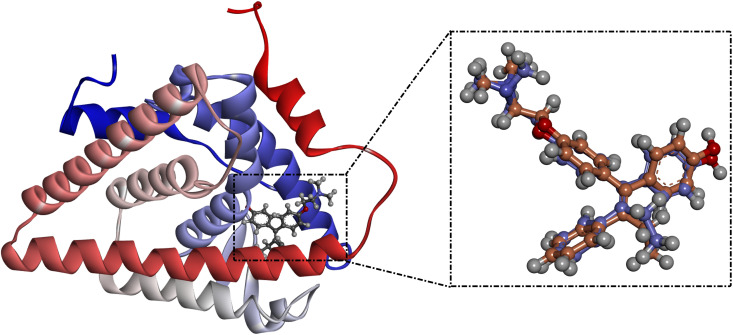
Overlapping of the anticipated docking mode (orange) and the original binding pose (mauve) of 4-hydroxytamoxifen with ER*α.*

To examine the docking pose of DAMNI with ER*α* and EGFR active sites, docking computations were executed. Assessment of the AutoDock4.2.6 software utilizing the specified parameters was first performed in alignment with the available experimental data. The co-crystallized ligand, namely 4-hydroxytamoxifen with the ER*α*, was subjected to redocking and subsequently compared to the original structure (PDB code: 3ERT) (Fig 9). As illustrated in Fig 9, the anticipated docking mode of 4-hydroxytamoxifen with ER*α* was nearly identical to the co-crystallized pose, displaying an RMSD value of 0.82 Å. In summary, the employed docking protocol can be used to predict the correct binding poses of ligands with the targeted receptors.

**Fig 10:**
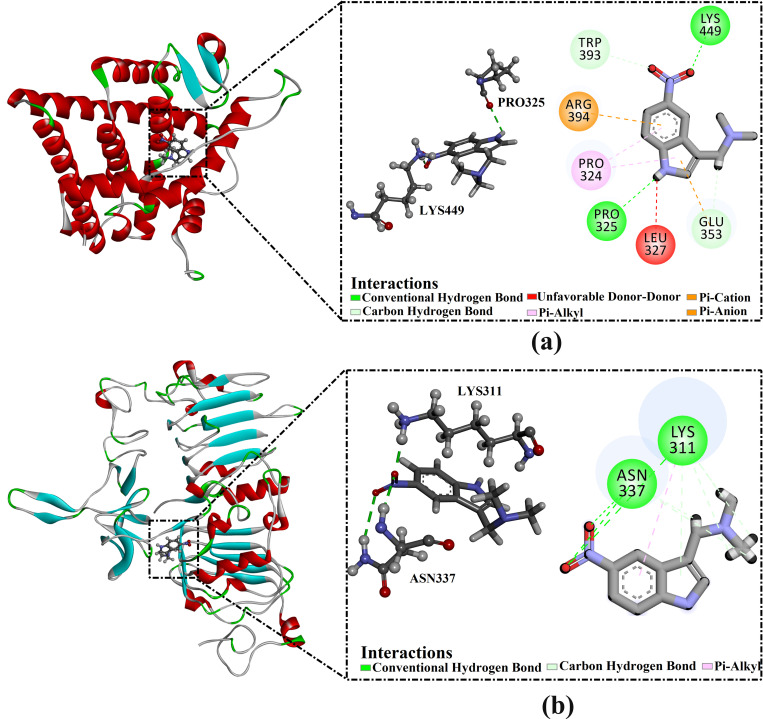
Three- and two-dimensional representations of the predicted docking modes of DAMNI within (a) ER*α* and (b) EGFR active sites.

DAMNI demonstrated better binding affinity against ER*α* compared to DAMNI towards EGFR with values of −5.8 and −4.7 kcal/mol, respectively. Investigating the docking mode of DAMNI within the ER*α* active site revealed that the NO_2_ exhibited an H-bond with LYS449 (1.78 Å) (Fig 10). Additionally, the NH of the pyrrole ring formed an H-bond with PRO325 (2.51 Å). On the other hand, analysis of the docking pose of DAMNI with EGFR active site unveiled that the NO_2_ group established three H-bonds with NH_2_ of ASN337 (2.68 Å), NH of ASN337 (2.11 Å), and NH_3_ of LYS311 (1.68 Å) (Fig 10). The presence of such H-bonds between the inhibitor and receptor resulted in strong inhibitor-receptor binding affinity [74].

### Molecular dynamics simulations (MDS)

Molecular dynamics simulations (MDS) are utilized to examine the time-independent interactions of an inhibitor bound to a receptor [75]. Accordingly, MDS was carried out for DAMNI complexed with ER*α* and EGFR over 150 ns, subsequently followed by evaluations of binding energy (Table 5). From Table 5, DAMNI-ER*α* displayed superior binding affinity compared to DAMNI-EGFR with Δ*G*_binding_ values of −21.1 and −15.4 kcal/mol, respectively. The estimated binding energies were subsequently decomposed into their components to elucidate the prevailing interactions between DAMNI and the investigated targets (Table 5). The *E*_vdW_ was identified as an important factor influencing the binding affinity of DAMNI to ER*α* and EGFR, with average values of −30.5 and −15.6 kcal/mol, respectively. The *E*_ele_ contribution to the Δ*G*_binding_ was favorable, with average values of −15.1 and −9.0 kcal/mol for DAMNI-ER*α* and DAMNI-EGFR complexes, respectively. Generally, DAMNI may be considered as a prospective inhibitor targeting ER*α* and EGFR for treating breast cancer.

**Table 5:** The estimated Δ*G*_binding_ and its components for DAMNI-ER*α* and DAMNI-EGFR complexes over the 150 ns MDS.

Compound Name	MM/GBSA Binding Energy (kcal/mol)						
	Δ*E*_*v*dW_	Δ*E*_ele_	Δ*E*_GB_	Δ*E*_SUR_	Δ*G*_gas_	Δ*G*_solv_	Δ*G*_binding_
DAMNI-ER*α*	−30.5	−15.1	28.5	−4.0	−45.6	24.6	−21.1
DAMNI-EGFR	−15.6	−9.0	10.7	−1.5	−24.6	9.3	−15.4

**Fig 11:**
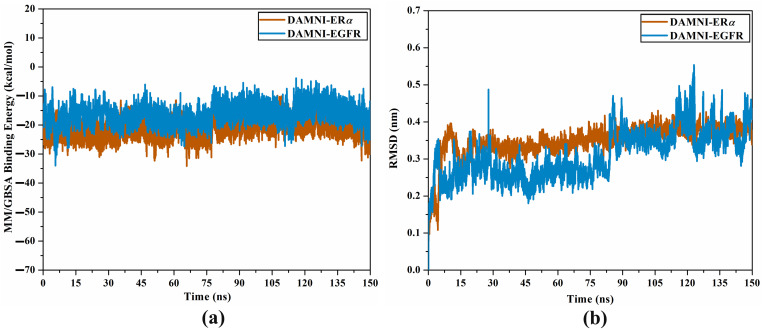
(a) Binding energy per-trajectory and (b) RMSD of the backbone atoms with respect to the starting structures of DAMNI-ER*α* (orange) and DAMNI-EGFR (light blue) complexes over the 150 ns MDS.

The energetic stability of DAMNI bound to ER*α* and EGFR was assessed by examining the correlation between binding energy and time (Fig 11a). As depicted in Fig 11a, this analysis revealed that all complexes under investigation exhibited energetic stability throughout the 150 ns MDS.

The RMSD analysis was employed to examine the conformational changes concerning inhibitor-receptor complexes throughout the simulated duration. Fig 11b illustrates the RMSD of DAMNI-ER*α* and DAMNI-EGFR complexes over the course of the 150 ns MDS. All complexes demonstrated overall stability following the initial 15 ns, at which point the conformational stability was realized. DAMNI maintained stability at its designated active sites during the simulated period, resulting in RMSD values below 0.5 nm. Overall, these findings indicated that DAMNI was firmly attached and did not alter the topology of the investigated targets.

**Fig 12:**
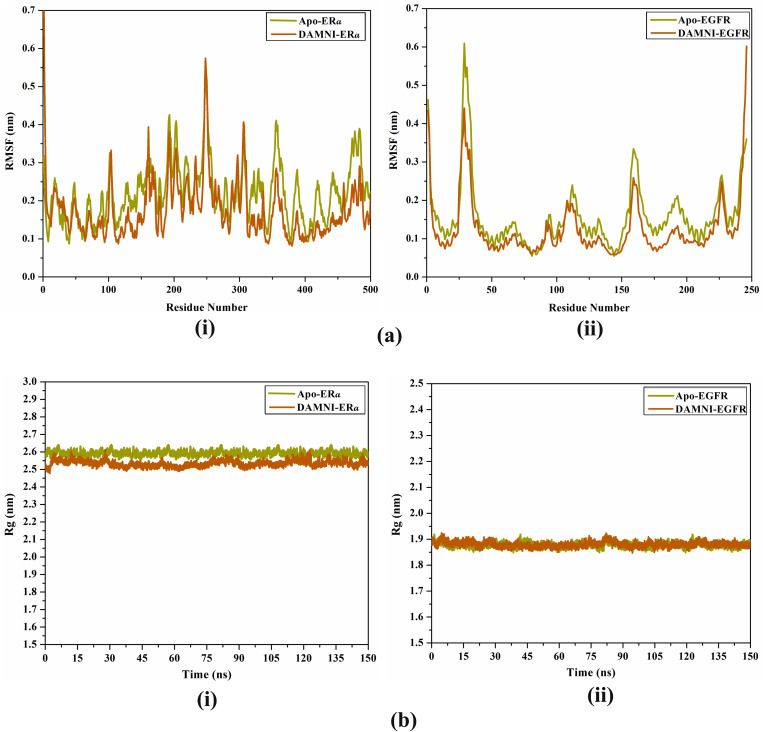
Evaluated (a) RMSF and (b) Rg for apo-form (yellow) and soaked ligand receptor (orange) for (i) ER*α* and (ii) EGFR over the 150 ns MDS.

To examine the differences in backbone structure and the stability of ER*α* and EGFR in both their unbound state and in complex with DAMNI, the root mean square fluctuation (RMSF) of C_*α*_ atoms was calculated and is presented in Fig 12a. As depicted in Fig 12a, the residues in the DAMNI-ER*α* and DAMNI-EGFR complexes exhibited relatively consistent behavior compared to the apo-receptor throughout the 150 ns MDS.

The radius of gyration (Rg) analysis was carried out to evaluate the compactness of ER*α* and EGFR in both their unbound state and in complex with DAMNI throughout 150 ns MDS. The Rg analysis offered valuable information regarding the overall folding and unfolding dynamics of the ER*α* and EGFR structures in the presence of DAMNI (Fig 12b). The mean Rg values recorded were 2.51, 2.58, 1.91, and 1.91 nm for the apo-ER*α*, DAMNI-ER*α*, apo-EGFR, and DAMNI-EGFR, respectively (Fig 12b). The Rg data suggested that ER*α* and EGFR retained their compact form upon the binding with DAMNI throughout the 150 ns MDS. These results revealed that the binding of DAMNI notably enhanced the stability of the ER*α* structure.

**Fig 13:**
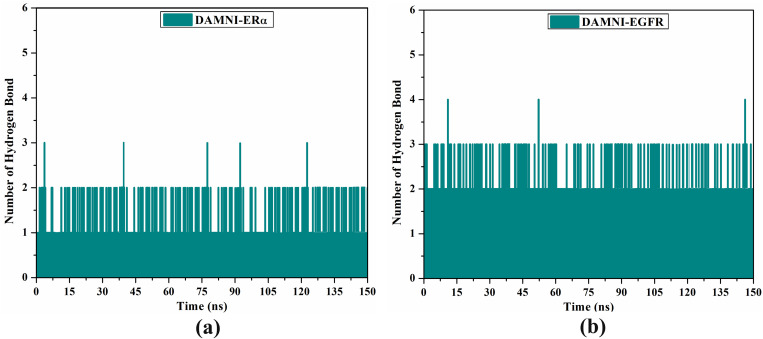
Evaluated H-bond number between DAMNI in complex with (a) ER*α* and (b) EGFR over the 150 ns MDS.

Since the formation of stable enzyme-inhibitor complexes relies on H-bonds, the number of intermolecular H-bonds formed between each DAMNI-ER*α* and DAMNI-EGFR complexes was assessed during a duration of 150 ns MDS. Fig 13 illustrates the number of H-bonds established between DAMNI complexed with ER*α* and DAMNI complexed with EGFR. The average number of H-bonds of the DAMNI-ER*α* and DAMNI-EGFR complexes was 2 and 3 throughout the 150 ns MDS. In general, these findings indicated a significant consistency with the expected docking illustrated in Fig 10.

## Conclusion

This study utilized DFT to examine the impact of polar protic and aprotic solvents on the molecular structure, vibrational properties, thermodynamic behavior, nonlinear optical characteristics, and electronic features of DAMNI. Additionally, molecular docking and MDS were executed to assess the molecule’s inhibitory potential on targeted receptors associated with breast cancer. The calculated first-order hyperpolarizability suggested that DAMNI could be a promising candidate for NLO applications. The analysis of the DOS and PDOS supported the FMOs analysis, highlighting charge transfer interactions within DAMNI. The reactive sites of the molecule were confirmed using local reactivity descriptors and the MEP surface. Molecular docking analysis showed that DAMNI had a promising binding affinity for ER*α* and EGFR, key receptors in breast cancer, with values of −5.8 and −4.7 kcal/mol, respectively. To examine receptor-ligand steadiness and conformational changes, those docking complexes were further subjected to 150 ns MDS. Based on the MM/GBSA//150 ns MDS, DAMNI-ER*α* displayed superior binding affinity compared to DAMNI-EGFR with Δ*G*_binding_ values of −21.1 and −15.4 kcal/mol, respectively. These findings hold promise for advancing effective breast cancer treatments and could offer valuable insights into the reactivity of biomolecules under solvent influence.

## Supporting information

S1 Table PES scans of DAMNI involving H17-C16-C1-C2 (Scan1) and C20-N19-C16-C1 (Scan2) dihedral angles in polar protic and aprotic solvents. (DOCX)

S2 Table Geometrical parameters of DAMNI in polar protic and aprotic solvents. (DOCX)

S3 Table Second-order perturbation analysis of Fock matrix in NBO basis of DAMNI in polar protic (ethanol) and aprotic solvents. (DOCX)

S4 Table Thermodynamic properties of DAMNI in polar protic and aprotic solvents. (DOCX)

S5 Table Vibrational frequency (cm^-1^) assignments of DAMNI in polar protic and aprotic solvents. (DOCX)

S1 Fig Theoretical FT-IR and FT-Raman spectra of DAMNI in (a) DMSO, (b) ethanol, and (c) acetone. (DOCX)
