# Supplementary material for: Insights into impact of polar protic and aprotic solvents on bioactive features of 3-(Dimethylaminomethyl)-5-nitroindole: A DFT study and molecular dynamics simulations
Source: PLoS One. 2025 Sep 10;20(9):e0330941. doi: 10.1371/journal.pone.0330941 (PMC12422483; doi:10.1371/journal.pone.0330941)
Supplement: S1 Table — (DOCX) [file pone.0330941.s001.docx]

**S1 Table.** PES scans of DAMNI involving H17-C16-C1-C2 (Scan1) and C20-N19-C16-C1 (Scan2) dihedral angles in polar protic and aprotic solvents.

| **Angles** | **Scan1** | | | | **Scan2** | | | |
| --- | --- | --- | --- | --- | --- | --- | --- | --- |
|  | **DMSO** | **Water** | **Ethanol** | **Acetone** | **DMSO** | **Water** | **Ethanol** | **Acetone** |
| 0 | −741.65067 | −741.65092 | −741.65012 | −741.64987 | −741.65065 | −741.65090 | −741.65010 | −741.64986 |
| 10 | −741.65051 | −741.65077 | −741.64996 | −741.64972 | −741.65006 | −741.65031 | −741.64952 | −741.64928 |
| 20 | −741.65017 | −741.65042 | −741.64961 | −741.64936 | −741.64898 | −741.64923 | −741.64844 | −741.64820 |
| 30 | −741.64960 | −741.64986 | −741.64904 | −741.64879 | −741.64778 | −741.64803 | −741.64725 | −741.64701 |
| 40 | −741.64880 | −741.64907 | −741.64823 | −741.64798 | −741.64692 | −741.64717 | −741.64639 | −741.64615 |
| 50 | −741.64794 | −741.64820 | −741.64735 | −741.64709 | −741.64683 | −741.64707 | −741.64629 | −741.64605 |
| 60 | −741.64708 | −741.64735 | −741.64648 | −741.64621 | −741.64757 | −741.64782 | −741.64704 | −741.64680 |
| 70 | −741.64648 | −741.64676 | −741.64587 | −741.64560 | −741.64889 | −741.64914 | −741.64835 | −741.64811 |
| 80 | −741.64674 | −741.64702 | −741.64612 | −741.64584 | −741.65008 | −741.65033 | −741.64953 | −741.64929 |
| 90 | −741.64788 | −741.64816 | −741.64726 | −741.64698 | −741.65067 | −741.65093 | −741.65013 | −741.64988 |
| 100 | −741.64921 | −741.64950 | −741.64860 | −741.64832 | −741.65099 | −741.65126 | −741.65042 | −741.65016 |
| 110 | −741.65045 | −741.65073 | −741.64983 | −741.64956 | −741.65028 | −741.65055 | −741.64971 | −741.64945 |
| 120 | −741.65141 | −741.65169 | −741.65080 | −741.65052 | −741.64881 | −741.64908 | −741.64824 | −741.64798 |
| 130 | −741.65207 | −741.65235 | −741.65147 | −741.65119 | −741.64689 | −741.64715 | −741.64631 | −741.64605 |
| 140 | −741.65243 | −741.65270 | −741.65183 | −741.65156 | −741.64490 | −741.64516 | −741.64432 | −741.64406 |
| 150 | −741.65247 | −741.65274 | −741.65188 | −741.65161 | −741.64319 | −741.64345 | −741.64261 | −741.64235 |
| 160 | −741.65210 | −741.65237 | −741.65152 | −741.65126 | −741.64223 | −741.64250 | −741.64165 | −741.64139 |
| 170 | −741.65140 | −741.65166 | −741.65083 | −741.65057 | −741.64228 | −741.64255 | −741.64169 | −741.64143 |
| 180 | −741.65025 | −741.65051 | −741.64969 | −741.64944 | −741.64330 | −741.64358 | −741.64271 | −741.64245 |
| 190 | −741.64887 | −741.64913 | −741.64831 | −741.64805 | −741.64501 | −741.64528 | −741.64441 | −741.64415 |
| 200 | −741.64732 | −741.64758 | −741.64675 | −741.64649 | −741.64703 | −741.64731 | −741.64643 | −741.64617 |
| 210 | −741.64567 | −741.64594 | −741.64510 | −741.64484 | −741.64880 | −741.64908 | −741.64820 | −741.64794 |
| 220 | −741.64420 | −741.64447 | −741.64362 | −741.64335 | −741.64990 | −741.65018 | −741.64930 | −741.64903 |
| 230 | −741.64348 | −741.64375 | −741.64289 | −741.64263 | −741.65010 | −741.65038 | −741.64950 | −741.64924 |
| 240 | −741.64974 | −741.65000 | −741.64917 | −741.64892 | −741.64934 | −741.64961 | −741.64874 | −741.64847 |
| 250 | −741.65096 | −741.65123 | −741.65040 | −741.65014 | −741.64773 | −741.64801 | −741.64714 | −741.64687 |
| 260 | −741.65183 | −741.65210 | −741.65125 | −741.65100 | −741.64563 | −741.64590 | −741.64503 | −741.64477 |
| 270 | −741.65235 | −741.65262 | −741.65176 | −741.65150 | −741.64348 | −741.64375 | −741.64289 | −741.64262 |
| 280 | −741.65249 | −741.65277 | −741.65190 | −741.65163 | −741.64195 | −741.64222 | −741.64135 | −741.64109 |
| 290 | −741.65227 | −741.65255 | −741.65167 | −741.65140 | −741.64163 | −741.64190 | −741.64103 | −741.64076 |
| 300 | −741.65175 | −741.65204 | −741.65114 | −741.65087 | −741.64275 | −741.64303 | −741.64215 | −741.64188 |
| 310 | −741.65089 | −741.65117 | −741.65027 | −741.65000 | −741.64499 | −741.64527 | −741.64438 | −741.64411 |
| 320 | −741.64975 | −741.65003 | −741.64913 | −741.64885 | −741.64762 | −741.64790 | −741.64702 | −741.64675 |
| 330 | −741.64840 | −741.64869 | −741.64778 | −741.64750 | −741.65003 | −741.65031 | −741.64943 | −741.64916 |
| 340 | −741.64705 | −741.64734 | −741.64643 | −741.64615 | −741.65172 | −741.65199 | −741.65112 | −741.65086 |
| 350 | −741.64646 | −741.64674 | −741.64585 | −741.64557 | −741.65248 | −741.65275 | −741.65188 | −741.65162 |
| 360 | −741.64687 | −741.64715 | −741.64628 | −741.64601 | −741.65233 | −741.65261 | −741.65174 | −741.65147 |
