# Supplementary material for: Insights into impact of polar protic and aprotic solvents on bioactive features of 3-(Dimethylaminomethyl)-5-nitroindole: A DFT study and molecular dynamics simulations
Source: PLoS One. 2025 Sep 10;20(9):e0330941. doi: 10.1371/journal.pone.0330941 (PMC12422483; doi:10.1371/journal.pone.0330941)
Supplement: S2 Table — (DOCX) [file pone.0330941.s002.docx]

**S2 Table.** Geometrical parameters of DAMNI in polar protic and aprotic solvents.

| **Geometrical parameters** | **Water** | **Ethanol** | **Acetone** | **DMSO** | **Geometrical parameters** | **Water** | **Ethanol** | **Acetone** | **DMSO** |
| --- | --- | --- | --- | --- | --- | --- | --- | --- | --- |
| **Bond Length (Å)** | | | | | **Bond Angle (°)** | | | | |
| N4-H13 | 1.007 | 1.007 | 1.007 | 1.007 | C1-C2-N4 | 110.19 | 110.18 | 110.18 | 110.19 |
| C2-H15 | 1.076 | 1.076 | 1.076 | 1.076 | C5-C7-C11 | 117.91 | 117.91 | 117.91 | 117.91 |
| C6-H9 | 1.079 | 1.079 | 1.079 | 1.079 | H13-N4-C2 | 124.96 | 124.96 | 124.96 | 124.96 |
| C7-H10 | 1.081 | 1.081 | 1.081 | 1.081 | H15-C2-C1 | 129.57 | 129.55 | 129.54 | 129.57 |
| C11-H12 | 1.078 | 1.078 | 1.078 | 1.078 | H9-C6-C3 | 121.90 | 121.92 | 121.93 | 121.91 |
| C20-H21 | 1.090 | 1.090 | 1.090 | 1.090 | H10-C7-C5 | 121.18 | 121.19 | 121.20 | 121.18 |
| C20-H23 | 1.090 | 1.090 | 1.090 | 1.090 | H12-C11-C7 | 120.71 | 120.73 | 120.73 | 120.71 |
| C24-H26 | 1.090 | 1.090 | 1.090 | 1.090 | H21-C20-C23 | 108.45 | 108.44 | 108.44 | 108.45 |
| C24-H27 | 1.089 | 1.089 | 1.089 | 1.089 | H23-C20-H22 | 108.17 | 108.17 | 108.17 | 108.17 |
| C16-H17 | 1.103 | 1.103 | 1.103 | 1.103 | H26-C24-H25 | 108.18 | 108.18 | 108.18 | 108.18 |
| C16-H18 | 1.093 | 1.093 | 1.093 | 1.093 | H27-C24-H26 | 108.33 | 108.33 | 108.33 | 108.33 |
| C20-C22 | 1.103 | 1.103 | 1.103 | 1.103 | H17-C16-C1 | 109.30 | 109.31 | 109.31 | 109.31 |
| C24-H25 | 1.102 | 1.103 | 1.103 | 1.103 | H18-C16-H17 | 105.93 | 105.93 | 105.93 | 105.93 |
| C1-C2 | 1.367 | 1.367 | 1.367 | 1.367 | H22-C20-H21 | 107.90 | 107.90 | 107.89 | 107.90 |
| O28-N14 | 1.231 | 1.231 | 1.231 | 1.231 | H25-C24-C27 | 108.20 | 108.19 | 108.19 | 108.20 |
| O29-N14 | 1.231 | 1.231 | 1.231 | 1.231 | O28-N14-C8 | 118.90 | 118.86 | 118.85 | 118.89 |
| C7-C11 | 1.378 | 1.378 | 1.378 | 1.378 | O29-N14−O28 | 122.43 | 122.50 | 122.52 | 122.45 |
| C3-C6 | 1.391 | 1.392 | 1.392 | 1.392 | C7-C11-C8 | 119.87 | 119.87 | 119.87 | 119.87 |
| C6-C8 | 1.389 | 1.389 | 1.389 | 1.389 | C3-C6-C8 | 117.88 | 117.88 | 117.88 | 117.88 |
| C5-C7 | 1.398 | 1.398 | 1.398 | 1.398 | C6-C8-C11 | 122.97 | 122.97 | 122.97 | 122.97 |
| N4-C5 | 1.359 | 1.359 | 1.359 | 1.359 | N4-C5-C3 | 107.11 | 107.11 | 107.11 | 107.11 |
| C11-C8 | 1.409 | 1.408 | 1.408 | 1.408 | C2-N4-C5 | 109.50 | 109.50 | 109.49 | 109.50 |
| N4-C2 | 1.382 | 1.382 | 1.382 | 1.382 | C16-C1-C2 | 127.51 | 127.48 | 127.47 | 127.50 |
| C16-C1 | 1.501 | 1.501 | 1.501 | 1.501 | N14-C8-C6 | 118.51 | 118.51 | 118.51 | 118.51 |
| N14-C8 | 1.452 | 1.453 | 1.453 | 1.452 | C24-N19-C20 | 110.57 | 110.59 | 110.59 | 110.58 |
| N19-C20 | 1.459 | 1.459 | 1.459 | 1.459 | C16-N19-C20 | 110.98 | 111.01 | 111.02 | 110.99 |
| N19-C24 | 1.460 | 1.460 | 1.460 | 1.460 | N19-C16-C1 | 113.53 | 113.52 | 113.51 | 113.53 |
| N19-C16 | 1.464 | 1.464 | 1.464 | 1.464 |  |  |  |  |  |
