# Supplementary material for: Insights into impact of polar protic and aprotic solvents on bioactive features of 3-(Dimethylaminomethyl)-5-nitroindole: A DFT study and molecular dynamics simulations
Source: PLoS One. 2025 Sep 10;20(9):e0330941. doi: 10.1371/journal.pone.0330941 (PMC12422483; doi:10.1371/journal.pone.0330941)
Supplement: S3 Table — (DOCX) [file pone.0330941.s003.docx]

**S3 Table.** Second-order perturbation analysis of Fock matrix in NBO basis of DAMNI in polar protic and aprotic solvents.

| **Ethanol** | | | | | | | |
| --- | --- | --- | --- | --- | --- | --- | --- |
| **No.** | **Donor NBO (i)** | **ED (e)** | **Acceptor NBO (j)** | **ED (e)** | ***E* (2) (Kcal/mol)** | ***E* (j) −*E* (i) (au)** | **F(i,j)**  **(au)** |
| 1. | σ (C1 – C2) | 1.97558 | σ*(C3 – C6) | 0.02019 | 6.45 | 1.25 | 0.080 |
| 2. | σ (C1 – C2) | 1.97558 | π*(C3 – C5) | 0.51464 | 16.61 | 0.28 | 0.067 |
| 3. | σ (C2 – N4) | 1.98447 | σ*(C5 – C7) | 0.02075 | 5.45 | 1.32 | 0.076 |
| 4. | σ (C3 – C5) | 1.96246 | σ*(C1 – C16) | 0.02345 | 5.49 | 1.06 | 0.068 |
| 5. | π (C3 − C5) | 1.52936 | π*(C1 – C2) | 0.27556 | 17.42 | 0.28 | 0.066 |
| 6. | π (C3 − C5) | 1.52936 | π*(C6 – C8) | 0.39008 | 25.98 | 0.26 | 0.075 |
| 7. | π (C3 − C5) | 1.52936 | π*(C7– C11) | 0.28416 | 16.35 | 0.27 | 0.062 |
| 8. | σ (C6 – C8) | 1.97601 | σ*(C1 – C3) | 0.02487 | 5.14 | 1.22 | 0.071 |
| 9. | π (C6 – C8) | 1.67959 | π*(C3– C5) | 0.51464 | 14.38 | 0.29 | 0.060 |
| 10. | π (C6 – C8) | 1.67959 | π*(C7– C11) | 0.28416 | 19.38 | 0.29 | 0.068 |
| 11. | π (C6 – C8) | 1.67959 | π*(N14– O29) | 0.65361 | 37.51 | 0.13 | 0.068 |
| 12. | σ (C6 – H9) | 1.97427 | σ*(C8 – C11) | 0.02230 | 5.01 | 1.03 | 0.064 |
| 13. | σ (C7 – H10) | 1.97407 | σ*(C3– C5) | 0.02915 | 5.22 | 1.01 | 0.065 |
| 14. | σ (C7 – C11) | 1.97532 | σ*(N4 – C5) | 0.02357 | 5.78 | 1.17 | 0.073 |
| 15. | π (C7 – C11) | 1.73647 | π*(C3– C5) | 0.51464 | 19.20 | 0.28 | 0.070 |
| 16. | π (C7 – C11) | 1.73647 | π* (C6– C8) | 0.39008 | 16.75 | 0.28 | 0.063 |
| 17. | σ (C11 – H12) | 1.97273 | σ*(C6– C8) | 0.01945 | 5.12 | 1.05 | 0.066 |
| 18. | π (N14 – O29) | 1.98733 | LP (3) O28 | 1.49085 | 10.97 | 0.17 | 0.075 |
| 19. | π (N14 – O29) | 1.98733 | π* (N14– O29) | 0.65361 | 7.75 | 0.31 | 0.053 |
| 20. | σ (C16 – H18) | 1.96655 | π*(C1– C2) | 0.27556 | 5.22 | 0.52 | 0.049 |
| 21. | LP (1) N4 | 1.58945 | π* (C1– C2) | 0.27556 | 33.12 | 0.30 | 0.091 |
| 22. | LP (1) N4 | 1.58945 | π*(C3– C5) | 0.51464 | 43.19 | 0.28 | 0.100 |
| 23. | LP (1) N19 | 1.87567 | σ*(C16 – H17) | 0.03700 | 7.50 | 0.66 | 0.064 |
| 24. | LP (1) N19 | 1.87567 | σ*(C20 – H22) | 0.03036 | 7.76 | 0.66 | 0.065 |
| 25. | LP (1) N19 | 1.87567 | σ*(C24 – H25) | 0.02973 | 7.57 | 0.66 | 0.064 |
| 26. | LP (2) O28 | 1.90370 | σ*(C8 – N14) | 0.10197 | 13.53 | 0.58 | 0.080 |
| 27. | LP (2) O28 | 1.90370 | σ*(N14 – O29) | 0.05727 | 19.04 | 0.70 | 0.104 |
| 28. | LP (2) O29 | 1.90464 | σ*(C8 – N14) | 0.10197 | 13.38 | 0.59 | 0.079 |
| 29. | LP (2) O29 | 1.90464 | σ*(N14 – O28) | 0.05724 | 19.05 | 0.70 | 0.104 |
|  | **Acetone** | | | | | | |
| 1. | σ (C1 – C2) | 1.97557 | σ*(C3 – C6) | 0.02019 | 6.44 | 1.25 | 0.080 |
| 2. | π (C1 – C2) | 1.82932 | π*(C3 – C5) | 0.51447 | 16.61 | 0.28 | 0.067 |
| 3. | σ (C2 – N4) | 1.98448 | σ*(C5 – C7) | 0.02076 | 5.45 | 1.32 | 0.076 |
| 4. | σ (C3 – C5) | 1.96245 | σ*(C1 – C16) | 0.02346 | 5.49 | 1.06 | 0.068 |
| 5. | π (C3 − C5) | 1.52974 | π* (C1 – C2) | 0.27564 | 17.43 | 0.28 | 0.066 |
| 6. | π (C3 − C5) | 1.52974 | π* (C6 – C8) | 0.38969 | 25.94 | 0.26 | 0.075 |
| 7. | π (C3 − C5) | 1.52974 | π* (C7– C11) | 0.28438 | 16.36 | 0.27 | 0.062 |
| 8. | σ (C6 – C8) | 1.97602 | σ*(C1 – C3) | 0.02487 | 5.14 | 1.22 | 0.071 |
| 9. | π (C6 – C8) | 1.67985 | π* (C3– C5) | 0.51447 | 14.38 | 0.29 | 0.060 |
| 10. | π (C6 – C8) | 1.67985 | π* (C7– C11) | 0.28438 | 19.40 | 0.29 | 0.068 |
| 11. | π (C6 – C8) | 1.67985 | π*(N14– O29) | 0.65317 | 37.37 | 0.13 | 0.068 |
| 12. | σ (C6 – C9) | 1.97427 | σ*(C8 – C11) | 0.02230 | 5.01 | 1.03 | 0.064 |
| 13. | σ (C7 – H10) | 1.97409 | σ* (C3– C5) | 0.02915 | 5.22 | 1.01 | 0.065 |
| 14. | σ (C7 – C11) | 1.97409 | σ*(N4 – C5) | 0.02358 | 5.79 | 1.17 | 0.073 |
| 15. | π (C7 – C11) | 1.73638 | π* (C3– C5) | 0.51447 | 19.21 | 0.28 | 0.070 |
| 16. | π (C7 – C11) | 1.73638 | π* (C6– C8) | 0.38969 | 16.75 | 0.28 | 0.063 |
| 17. | σ (C11 – H12) | 1.97272 | σ* (C6– C8) | 0.01945 | 5.12 | 1.05 | 0.066 |
| 18. | π (N14 – O29) | 1.98732 | LP (3) O28 | 1.49041 | 10.98 | 0.17 | 0.075 |
| 19. | π (N14 – O29) | 1.98732 | π* (N14– O29) | 0.65317 | 7.74 | 0.31 | 0.053 |
| 20. | σ (C16 – H18) | 1.96655 | π*(C1– C2) | 0.27564 | 5.22 | 0.52 | 0.049 |
| 21. | LP (1) N4 | 1.58991 | π*(C1– C2) | 0.27564 | 33.11 | 0.30 | 0.091 |
| 22. | LP (1) N4 | 1.58991 | π*(C3– C5) | 0.51447 | 43.13 | 0.28 | 0.100 |
| 23. | LP (1) N19 | 1.87561 | σ*(C16 – H17) | 0.03701 | 7.50 | 0.66 | 0.064 |
| 24. | LP (1) N19 | 1.87561 | σ*(C20 – H22) | 0.03037 | 7.76 | 0.66 | 0.065 |
| 25. | LP (1) N19 | 1.87561 | σ*(C24 – H25) | 0.02974 | 7.58 | 0.66 | 0.064 |
| 26. | LP (2) O28 | 1.90361 | σ*(C8 – N14) | 0.10211 | 13.55 | 0.58 | 0.080 |
| 27. | LP (2) O28 | 1.90361 | σ*(N14 – O29) | 0.05727 | 19.04 | 0.70 | 0.104 |
| 28. | LP (2) O29 | 1.90455 | σ*(C8 – N14) | 0.10211 | 13.40 | 0.58 | 0.079 |
| 29. | LP (2) O29 | 1.90455 | σ*(N14 – O28) | 0.05725 | 19.05 | 0.70 | 0.104 |
|  | **DMSO** | | | | | | |
| 1. | σ (C1 – C2) | 1.97559 | σ*(C3 – C6) | 0.02018 | 6.45 | 1.25 | 0.080 |
| 2. | π (C1 – C2) | 1.82894 | π*(C3 – C5) | 0.51502 | 16.62 | 0.28 | 0.067 |
| 3. | σ (C2 – N4) | 1.98446 | σ*(C5 – C7) | 0.02075 | 5.46 | 1.32 | 0.076 |
| 4. | σ (C3 – C5) | 1.96249 | σ*(C1 – C16) | 0.02345 | 5.49 | 1.06 | 0.068 |
| 5. | π (C3 − C5) | 1.52851 | π* (C1 – C2) | 0.27539 | 17.40 | 0.28 | 0.066 |
| 6. | π (C3 − C5) | 1.52851 | π* (C6 – C8) | 0.39097 | 26.06 | 0.26 | 0.075 |
| 7. | π (C3 − C5) | 1.52851 | π* (C7– C11) | 0.28367 | 16.33 | 0.27 | 0.062 |
| 8. | σ (C6 – C8) | 1.97599 | σ*(C1 – C3) | 0.02488 | 5.14 | 1.22 | 0.071 |
| 9. | π (C6 – C8) | 1.67898 | π* (C3– C5) | 0.51502 | 14.36 | 0.29 | 0.060 |
| 10. | π (C6 – C8) | 1.67898 | π* (C7– C11) | 0.28367 | 19.34 | 0.29 | 0.068 |
| 11. | π (C6 – C8) | 1.67898 | π*(N14– O29) | 0.65460 | 37.83 | 0.13 | 0.068 |
| 12. | σ (C6 – C9) | 1.97428 | σ*(C8 – C11) | 0.02230 | 5.01 | 1.03 | 0.064 |
| 13. | σ (C7 – H10) | 1.97405 | σ* (C3– C5) | 0.02916 | 5.22 | 1.01 | 0.065 |
| 14. | σ (C7 – C11) | 1.97534 | σ*(N4 – C5) | 0.02354 | 5.78 | 1.17 | 0.073 |
| 15. | π (C7 – C11) | 1.73666 | π* (C3– C5) | 0.51502 | 19.18 | 0.28 | 0.070 |
| 16. | π (C7 – C11) | 1.73666 | π* (C6– C8) | 0.39097 | 16.74 | 0.28 | 0.063 |
| 17. | σ (C11 – H12) | 1.97274 | σ* (C6– C8) | 0.01945 | 5.12 | 1.05 | 0.066 |
| 18. | π (N14 – O29) | 1.98736 | LP (3) O28 | 1.49184 | 10.94 | 0.17 | 0.075 |
| 19. | π (N14 – O29) | 1.98736 | π* (N14– O29) | 0.05726 | 7.76 | 0.31 | 0.053 |
| 20. | σ (C16 – H18) | 1.96653 | π*(C1– C2) | 0.27539 | 5.22 | 0.52 | 0.049 |
| 21. | LP (1) N4 | 1.58841 | π*(C1– C2) | 0.27539 | 33.14 | 0.30 | 0.092 |
| 22. | LP (1) N4 | 1.58841 | π*(C3– C5) | 0.51502 | 43.34 | 0.28 | 0.100 |
| 23. | LP (1) N19 | 1.87581 | σ*(C16 – H17) | 0.03697 | 7.49 | 0.66 | 0.064 |
| 24. | LP (1) N19 | 1.87581 | σ*(C20 – H22) | 0.03034 | 7.75 | 0.66 | 0.065 |
| 25. | LP (1) N19 | 1.87581 | σ*(C24 – H25) | 0.02970 | 7.57 | 0.66 | 0.064 |
| 26. | LP (2) O28 | 1.90391 | σ*(C8 – N14) | 0.10166 | 13.49 | 0.59 | 0.079 |
| 27. | LP (2) O28 | 1.90391 | σ*(N14 – O29) | 0.05726 | 19.02 | 0.70 | 0.104 |
| 28. | LP (2) O29 | 1.90485 | σ*(C8 – N14) | 0.10166 | 13.34 | 0.59 | 0.079 |
| 29. | LP (2) O29 | 1.90485 | σ*(N14 – O28) | 0.05724 | 19.03 | 0.70 | 0.104 |
