# Supplementary material for: Insights into impact of polar protic and aprotic solvents on bioactive features of 3-(Dimethylaminomethyl)-5-nitroindole: A DFT study and molecular dynamics simulations
Source: PLoS One. 2025 Sep 10;20(9):e0330941. doi: 10.1371/journal.pone.0330941 (PMC12422483; doi:10.1371/journal.pone.0330941)
Supplement: S4 Table — (DOCX) [file pone.0330941.s004.docx]

**S4 Table.** Thermodynamic properties of DAMNI in polar protic and aprotic solvents.

| **T (K)** | **Cp** | ***S*** | **Δ*H*** | **Cp** | ***S*** | **Δ*H*** | **Cp** | ***S*** | **Δ*H*** | **Cp** | ***S*** | **Δ*H*** |
| --- | --- | --- | --- | --- | --- | --- | --- | --- | --- | --- | --- | --- |
|  | **Water** | | | **Ethanol** | | | **Acetone** | | | **DMSO** | | |
| 100 | 100.283 | 337.919 | 6.643 | 100.298 | 337.797 | 6.642 | 100.303 | 337.760 | 6.642 | 100.289 | 337.885 | 6.643 |
| 200 | 168.026 | 428.254 | 20.063 | 168.063 | 428.152 | 20.065 | 168.075 | 428.122 | 20.066 | 168.038 | 428.227 | 20.064 |
| 298.15 | 236.617 | 508.045 | 39.901 | 236.633 | 507.954 | 39.906 | 236.638 | 507.928 | 39.907 | 236.622 | 508.021 | 39.903 |
| 300 | 237.921 | 509.513 | 40.340 | 237.937 | 509.422 | 40.345 | 237.942 | 509.396 | 40.346 | 237.927 | 509.489 | 40.342 |
| 400 | 306.007 | 587.431 | 67.588 | 305.999 | 587.342 | 67.593 | 305.997 | 587.316 | 67.595 | 306.005 | 587.408 | 67.590 |
| 500 | 365.258 | 662.275 | 101.239 | 365.236 | 662.182 | 101.243 | 365.230 | 662.156 | 101.244 | 365.251 | 662.251 | 101.241 |
| 600 | 414.086 | 733.336 | 140.289 | 414.059 | 733.239 | 140.290 | 414.051 | 733.211 | 140.291 | 414.078 | 733.311 | 140.290 |
| 700 | 453.939 | 800.263 | 183.757 | 453.910 | 800.161 | 183.755 | 453.902 | 800.132 | 183.755 | 453.930 | 800.236 | 183.757 |
| 800 | 486.736 | 863.088 | 230.842 | 486.710 | 862.983 | 230.838 | 486.701 | 862.952 | 230.836 | 486.728 | 863.060 | 230.841 |
| 900 | 514.047 | 922.042 | 280.922 | 514.022 | 921.933 | 280.915 | 514.014 | 921.902 | 280.913 | 514.039 | 922.013 | 280.920 |
| 1000 | 537.018 | 977.425 | 333.507 | 536.996 | 977.314 | 333.498 | 536.989 | 977.282 | 333.495 | 537.011 | 977.395 | 333.505 |

Cp and *S* in J/mol.K

Δ*H* in kJ/mol
