# Supplementary material for: Insights into impact of polar protic and aprotic solvents on bioactive features of 3-(Dimethylaminomethyl)-5-nitroindole: A DFT study and molecular dynamics simulations
Source: PLoS One. 2025 Sep 10;20(9):e0330941. doi: 10.1371/journal.pone.0330941 (PMC12422483; doi:10.1371/journal.pone.0330941)
Supplement: S5 Table — (DOCX) [file pone.0330941.s005.docx]

**S5 Table.** Vibrational frequency (cm^-1^) assignments of DAMNI in polar protic and aprotic solvents.

| **N°** | **IR** | **Raman** | **Scaled frequency in water** | | | **Scaled frequency in other solvents** | | | **Assignment (PED %)** |
| --- | --- | --- | --- | --- | --- | --- | --- | --- | --- |
|  |  |  | **Water** | **IR Intensity** | **Raman Activity** | **Ethanol** | **Αcetone** | **DMSO** |  |
| 1 | 3460 |  | 3495.83 | 259.1694 | 584.1102 | 3497.23 | 3497.67 | 3496.28 | ν NH (100) ring |
| 2 |  |  | 3137.93 | 0.8795 | 302.4707 | 3137.87 | 3137.86 | 3137.91 | ν CH (99) ring |
| 3 |  | 3105 | 3108.85 | 4.7507 | 275.2233 | 3108.84 | 3108.84 | 3108.85 | ν CH (100) ring |
| 4 |  | 3078 | 3100.74 | 2.8580 | 185.1619 | 3100.73 | 3100.73 | 3100.74 | ν CH (97) ring |
| 5 | 3053 | 3054 | 3071.89 | 4.6141 | 305.5678 | 3071.45 | 3071.32 | 3071.76 | ν CH (97) ring |
| 6 |  |  | 2987.25 | 44.5035 | 163.3626 | 2987.28 | 2987.29 | 2987.26 | ν_as_ CH_3_ (97) |
| 7 |  |  | 2978.34 | 45.5800 | 187.0254 | 2978.35 | 2978.35 | 2978.35 | ν_as_ CH_3_ (96) |
| 8 |  |  | 2945.18 | 51.6014 | 405.7633 | 2945.20 | 2945.21 | 2945.19 | ν_as_ CH_3_ (97) |
| 9 |  |  | 2937.99 | 51.7991 | 247.1063 | 2938.03 | 2938.05 | 2938.01 | ν_as_ CH_3_ (95) |
| 10 |  |  | 2911.88 | 37.0360 | 351.3404 | 2911.84 | 2911.84 | 2911.87 | ν_as_ CH_3_ (94) |
| 11 |  |  | 2810.80 | 324.3387 | 863.3490 | 2810.79 | 2810.79 | 2810.80 | ν CH (99) |
| 12 |  |  | 2805.87 | 65.8421 | 80.7550 | 2805.69 | 2805.64 | 2805.82 | ν CH (49) + ν CH (42) |
| 13 |  |  | 2801.48 | 61.2216 | 104.5762 | 2801.18 | 2801.09 | 2801.39 | ν CH (47) + ν CH (44) |
| 14 |  |  | 1589.04 | 63.6855 | 123.7550 | 1589.35 | 1589.45 | 1589.14 | ν CH (88) + ν CH (11) |
| 15 | 1578 | 1577 | 1546.21 | 28.7208 | 146.9286 | 1546.50 | 1546.58 | 1546.30 | ν CC (24) ring + ν CC (12) ring |
| 16 | 1517 | 1518 | 1519.95 | 22.7662 | 419.1647 | 1520.38 | 1520.51 | 1520.09 | ν CC (26) ring + ν CC (18) ring |
| 17 | 1456 | 1456 | 1474.60 | 213.4086 | 32.0928 | 1476.05 | 1476.50 | 1475.05 | ν CC (43) ring |
| 18 | 1436 | 1436 | 1440.32 | 11.9635 | 32.6457 | 1440.82 | 1440.97 | 1440.47 | ν_as_ NO_2_ (40) + ν CC (11) ring |
| 19 |  |  | 1433.13 | 22.9415 | 39.3375 | 1433.56 | 1433.70 | 1433.25 | *β*_as_CH_3_ (52) + *β*_as_CH_3_ (15) |
| 20 |  |  | 1432.83 | 109.6243 | 45.3685 | 1432.84 | 1432.86 | 1432.83 | *β*_as_CH_3_ (19) + *β*CH_2_ (14) + *β*_as_CH_3_ (10) |
| 21 |  |  | 1430.48 | 24.7957 | 3.8191 | 1430.89 | 1431.02 | 1430.60 | *β*_as_CH_3_ (20) + *β*_as_CH_3_ (10) |
| 22 |  |  | 1423.78 | 48.5492 | 13.8269 | 1424.47 | 1424.73 | 1423.96 | *β*_as_CH_3_ (29) + *β*_as_CH_3_ (26) |
| 23 |  |  | 1422.49 | 216.7457 | 14.8535 | 1423.49 | 1423.71 | 1422.85 | *β*_as_CH_3_ (30) + *β*_as_CH_3_ (23) |
| 24 | 1393 | 1393 | 1416.60 | 7.6759 | 24.1029 | 1417.00 | 1417.13 | 1416.73 | ν_as_ NO_2_ (31) |
| 25 |  |  | 1408.01 | 61.6035 | 59.3567 | 1408.33 | 1408.42 | 1408.11 | *β*_as_CH_3_ (46) + *β*_as_CH_3_ (16) |
| 26 |  |  | 1399.28 | 69.9088 | 144.1803 | 1399.37 | 1399.40 | 1399.31 | *β*_s_CH_3_ (19) + *β*CH_2_ (22) |
| 27 |  |  | 1381.13 | 1.1556 | 6.6383 | 1381.34 | 1381.40 | 1381.20 | *β*HNC (22) ring + *β*HCC (15) ring |
| 28 |  |  | 1361.14 | 33.9475 | 43.8693 | 1360.98 | 1360.93 | 1361.09 | *β*_s_CH_3_ (39) + *β*_s_CH_3_ (26) |
| 29 |  |  | 1333.67 | 42.4532 | 94.4654 | 1333.63 | 1333.62 | 1333.66 | τHCCC (18) + τHCCC (14) + ν CC (14) ring |
| 30 | 1295 | 1299 | 1295.43 | 38.4192 | 211.8721 | 1295.37 | 1295.36 | 1295.41 | νCC (19) ring + ν CC (14) ring |
| 31 |  |  | 1273.03 | 1539.397 | 3346.405 | 1275.10 | 1275.73 | 1273.69 | *β*HNC (13) ring |
| 32 |  |  | 1270.43 | 12.2783 | 47.1846 | 1270.27 | 1270.22 | 1270.38 | ν_s_NO_2_ (61) + *β*ONO (10) |
| 33 |  |  | 1247.65 | 1.8713 | 7.0407 | 1247.65 | 1247.65 | 1247.65 | *β*HCC (19) + *β*HNC (11) ring + τHCCC (12) |
| 34 | 1204 | 1246 | 1237.63 | 19.1981 | 3.2656 | 1237.86 | 1237.93 | 1237.70 | *β*HNC (20) ring |
| 35 |  |  | 1215.99 | 89.6275 | 264.2495 | 1215.92 | 1215.90 | 1215.97 | τHCNC (18) + τHCNC (13) + νNC (13) |
| 36 |  | 1208 | 1199.97 | 64.4951 | 293.2625 | 1199.92 | 1199.91 | 1199.96 | *β*HCC (17) + *β*HNC (11) + *β*HCC (11) ring |
| 37 | 1102 | 1105 | 1150.91 | 16.8805 | 16.4530 | 1151.06 | 1151.11 | 1150.95 | νNC (26) ring + *β*HNC (11) ring |
| 38 |  |  | 1129.43 | 8.4372 | 11.1837 | 1129.49 | 1129.50 | 1129.44 | νNC (17) + νNC (13) + τHCCC (13) |
| 39 |  |  | 1106.76 | 7.6148 | 2.6060 | 1106.83 | 1106.85 | 1106.78 | τHCNC (18) + τHCNC (18) + βHCC (10) |
| 40 | 1059 | 1059 | 1074.84 | 62.6788 | 90.5758 | 1074.99 | 1075.03 | 1074.89 | *β*HCC (27) ring + *β*HNC (25) ring +νCC (26) |
| 41 |  |  | 1072.13 | 215.5764 | 444.3797 | 1072.49 | 1072.59 | 1072.24 | τHCNC (19) + τHCNC (15) + τHCNC (13) |
| 42 |  |  | 1052.03 | 72.7590 | 29.0112 | 1052.07 | 1052.09 | 1052.05 | νNC (11) + νCC (10) |
| 43 |  |  | 1017.86 | 120.5668 | 131.5809 | 1018.10 | 1018.17 | 1017.94 | νNC (35) ring +*β*HCC (28) ring + *β*HCC (19) ring |
| 44 |  |  | 1012.52 | 35.8959 | 21.7553 | 1012.73 | 1012.80 | 1012.59 | νNC (12) + νNC (11) |
| 45 | 986 | 988 | 985.49 | 70.1912 | 13.8016 | 985.79 | 985.88 | 985.59 | νNC (16) ring + νNC (13) ring |
| 46 |  |  | 955.09 | 9.0906 | 21.5305 | 955.17 | 955.19 | 955.12 | νNC (28) + νNC (21) |
| 47 |  |  | 938.61 | 0.1270 | 0.4772 | 938.43 | 938.37 | 938.55 | *β*HCC (20) + τHCCC (14) + τNCCC (12) |
| 48 |  |  | 904.09 | 10.5932 | 36.7254 | 904.09 | 904.10 | 904.09 | τHCCN (45) ring + τHCCN (39) ring |
| 49 | 855 | 871 | 895.21 | 25.8241 | 2.8800 | 895.41 | 895.47 | 895.27 | *β*CNC (20) ring + *β*CCC (10) ring |
| 50 |  |  | 830.34 | 41.2957 | 17.0887 | 830.03 | 829.94 | 830.24 | τHCCC (76) ring |
| 51 |  |  | 824.93 | 14.9051 | 6.3324 | 824.94 | 824.94 | 824.93 | τHCCC (51) |
| 52 |  |  | 813.68 | 21.2905 | 20.0486 | 813.59 | 813.56 | 813.65 | *β*NO_2_ (18) +*β*CCC (17) ring +*β*CCC (17) ring |
| 53 | 798 | 799 | 801.30 | 23.9341 | 3.8474 | 801.31 | 801.31 | 801.31 | τHCCN (45) ring + νNC (19) + νNC (10) |
| 54 |  |  | 782.72 | 25.9573 | 118.3022 | 782.87 | 782.91 | 782.77 | τHCCN (28) ring + τHCCN (25) ring |
| 55 | 757 |  | 751.48 | 16.4741 | 2.4740 | 751.39 | 751.36 | 751.45 | *β*NO_2_ (25) + *β*CCC (13) ring |
| 56 |  |  | 734.31 | 47.8421 | 6.5163 | 734.34 | 734.34 | 734.32 | γNCCC (20) ring + γOCON (21) + τCCCC (25) ring |
| 57 | 728 | 729 | 728.99 | 13.7316 | 19.3002 | 729.02 | 729.03 | 729.00 | γOCON (39) + τHCCN (11) ring + τHCCN (10) ring |
| 58 | 657 | 658 | 658.83 | 38.2182 | 6.5143 | 658.82 | 658.81 | 658.83 | βCNC (12) ring |
| 59 | 623 | 621 | 607.39 | 1.5885 | 4.7206 | 607.37 | 607.37 | 607.38 | βNO_2_ (17) +βNCC (13) ring + βCCC (10) ring |
| 60 | 595 | 593 | 583.15 | 3.7925 | 14.0988 | 583.21 | 583.22 | 583.17 | τCCNC (41) ring + γCCCC (13) |
| 61 |  |  | 563.88 | 25.9305 | 1.1916 | 563.75 | 563.72 | 563.84 | βCCC (17) ring + νCC (10) ring |
| 62 | 542 | 543 | 536.60 | 15.1541 | 0.3851 | 536.51 | 536.49 | 536.58 | τCCCC (25) ring + γNCCC (15) ring |
| 63 |  | 501 | 493.21 | 106.4931 | 1.9081 | 490.51 | 489.68 | 492.35 | βONC (38) + βNCC (14) ring |
| 64 |  |  | 471.83 | 2.8253 | 3.1860 | 471.71 | 471.67 | 471.79 | τHNCC (85) |
| 65 |  |  | 415.40 | 2.3003 | 1.7041 | 415.37 | 415.36 | 415.39 | βONC (24) + βCNC (11) |
| 66 |  |  | 412.77 | 28.6152 | 1.1330 | 412.88 | 412.91 | 412.81 | βCNC (45) |
| 67 |  |  | 369.94 | 13.4683 | 0.8386 | 369.86 | 369.84 | 369.91 | τCCCC (50) ring + τCCCC (19) ring +τCNCC (17) ring |
| 68 |  |  | 344.71 | 1.5582 | 2.5559 | 344.59 | 344.55 | 344.67 | γCCCN (55) |
| 69 |  |  | 338.60 | 4.4281 | 0.2831 | 338.51 | 338.48 | 338.57 | βCNC (22) + νNC (20) |
| 70 |  |  | 324.51 | 3.1579 | 2.2025 | 324.45 | 324.43 | 324.49 | βCNC (26) + νNC (15) |
| 71 |  |  | 270.24 | 9.7943 | 0.6144 | 270.32 | 270.34 | 270.27 | γCCCC (21) ring + τCCCC (12) ring |
| 72 |  |  | 249.44 | 0.7308 | 1.7762 | 249.36 | 249.33 | 249.41 | γNCCC (19) ring +τCCCC (19) ring + γCCCN (16) ring |
| 73 |  |  | 247.48 | 2.3577 | 5.3303 | 247.42 | 247.40 | 247.46 | τHCNC (19) + βNCC (19) + τHCNC (16) |
| 74 |  |  | 240.19 | 0.8326 | 1.3578 | 240.14 | 240.12 | 240.17 | βNCC (14) + βCNC (10) |
| 75 |  |  | 185.20 | 1.0084 | 2.6036 | 185.18 | 185.17 | 185.19 | τHCNC (29) + τHCNC (23) + τHCNC (22) |
| 76 |  |  | 160.80 | 2.4818 | 4.5308 | 160.73 | 160.71 | 160.78 | βNCC (23) + βNCC (14) + τCNCC (14) ring |
| 77 |  |  | 110.01 | 0.6665 | 4.8461 | 110.13 | 110.16 | 110.05 | τCNCC (27) ring + τCNCC (12) + γNCCC (10) ring |
| 78 |  |  | 79.88 | 2.8523 | 5.3913 | 79.87 | 79.87 | 79.87 | γNCCC (27) ring + τCCCC (27) ring |
| 79 |  |  | 63.64 | 3.0835 | 2.1919 | 63.54 | 63.51 | 63.60 | βCCC (25) + βNCC (17) |
| 80 |  |  | 48.14 | 0.1061 | 5.2542 | 48.10 | 48.08 | 48.12 | τCNCC (59) + βCCC (11) |
| 81 |  |  | 21.62 | 1.1307 | 5.9370 | 21.99 | 22.11 | 21.73 | τONCC (80) |
