# Supplementary figures and images for: Insights into impact of polar protic and aprotic solvents on bioactive features of 3-(Dimethylaminomethyl)-5-nitroindole: A DFT study and molecular dynamics simulations
Source: PLoS One. 2025 Sep 10;20(9):e0330941. doi: 10.1371/journal.pone.0330941 (PMC12422483; doi:10.1371/journal.pone.0330941)

**
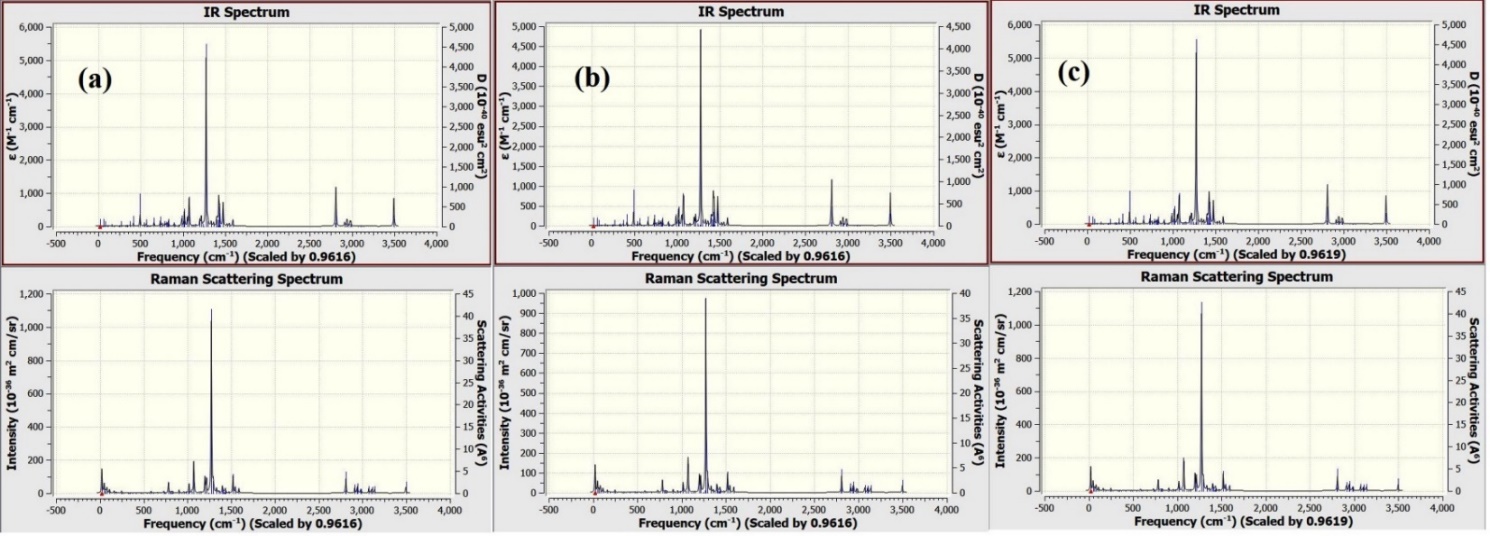
**

**S1 Fig.** Theoretical FT-IR and FT-Raman spectra of DAMNI in (a) DMSO, (b) ethanol, and (c) acetone.

Supplement: S1 Fig — (DOCX) [file pone.0330941.s006.docx]
